# Site-directed cation ordering in chabazite-type Al_*x*_Ga_1−*x*_PO_4_-34 frameworks revealed by NMR crystallography†

**DOI:** 10.1039/d3sc06924a

**Published:** 2024-02-14

**Authors:** Daniel M. Dawson, Jasmine A. Clayton, Thomas H. D. Marshall, Nathalie Guillou, Richard I. Walton, Sharon E. Ashbrook

**Affiliations:** a School of Chemistry, EaStCHEM and St Andrews Centre for Magnetic Resonance, University of St Andrews North Haugh St Andrews KY16 9ST UK dmd7@st-andrews.ac.uk sema@st-andrews.ac.uk; b Department of Chemistry, University of Warwick Coventry CV4 7AL UK R.I.Walton@warwick.ac.uk; c Institut Lavoisier, UMR CNRS 8180, Université de Versailles St-Quentin-en-Yvelines, Université Paris-Saclay 78035 Versailles France

## Abstract

We report the first synthesis of the mixed-metal chabazite-type Al_*x*_Ga_1−*x*_PO_4_-34(mim) solid solution, containing 1-methylimidazolium, mim, as structure directing agent (SDA), from the parent mixed-metal oxide solid solution, γ-(Al_*x*_Ga_1−*x*_)_2_O_3_. This hitherto unreported family of materials exhibits complex disorder, arising from the possible distributions of cations over available sites, the orientation of the SDA and the presence of variable amounts of water, which provides a prototype for understanding structural subtleties in nanoporous materials. In the as-made forms of the phosphate frameworks, there are three crystallographically distinct metal sites: two tetrahedral MO_4_ and one octahedral MO_4_F_2_ (M = Al, Ga). A combination of solid-state NMR spectroscopy and periodic DFT calculations reveals that the octahedral site is preferentially occupied by Al and the tetrahedral sites by Ga, leading to a non-random distribution of cations within the framework. Upon calcination to the Al_*x*_Ga_1−*x*_PO_4_-34 framework, all metal sites are tetrahedral and crystallographically equivalent in the average *R*3̄ symmetry. The cation distribution was explored by ^31^P solid-state NMR spectroscopy, and it is shown that the non-random distribution demonstrated to exist in the as-made materials would be expected to give remarkably similar patterns of peak intensities to a random distribution owing to the change in average symmetry in the calcined materials.

## Abstract 2

NMR crystallography exposes preferential site ordering of Al and Ga in new mixed-metal phosphate frameworks.

## Graphical abstract

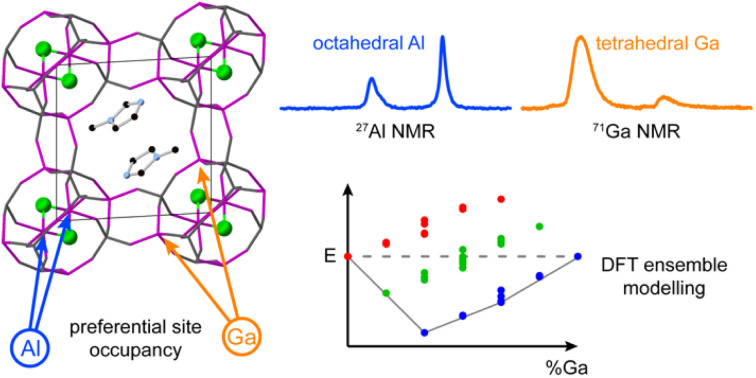

## Introduction

Since early studies of the preparation of synthetic microporous aluminophosphate frameworks (AlPOs) in the 1980s,^1^ there has been a long-standing interest in preparing examples of these zeolitic framework types, their structural characterisation, and investigating their properties (for representative reviews of the field, see *e.g.*, ref. 2–7). The gallophosphate analogues of AlPOs (GaPOs), have also been explored, and it has been demonstrated that some unique framework types can only (currently) be accessed as GaPOs,^8^ owing to subtle differences in the chemistry of Ga compared to Al. This makes GaPOs an appealing target of synthetic exploration despite their typically lower hydrothermal stability.^9^ Although there has been extensive work on both AlPOs and GaPOs, the mixed-metal AlGaPOs have only been mentioned three times in the literature; in a patent,^10^ a review article,^11^ and one report of the synthesis of cloverite-type AlGaPOs.^12^ Only for the cloverite materials were the synthetic details and limited structural characterisation reported, and AlGaPOs have apparently not been further studied since the 1990s. Indeed, in a more recent review drawing on the information within the International Zeolite Association database, AlGaPOs were not mentioned at all.^13^

It is well known that preparing mixed-metal forms of isostructural materials can lead to a combination of properties from the single-metal end members or, in some cases new or enhanced properties. In the context of phosphate frameworks, the important properties that may be affected by preparing mixed-metal AlGaPOs include molecular adsorption and diffusivity, thermal expansivity and catalytic activity. The distribution of the cations and any preferential site occupancy in mixed-metal materials can be challenging to determine by diffraction methods that probe the long-range average structure. However, these subtleties in the local structure are likely to strongly influence the properties of the material.

While Al^3+^ and Ga^3+^ each may occupy 4-, 5- or 6-coordinate sites in extended solids, in mixed-metal Al–Ga oxide materials, Ga^3+^ has a tendency for tetrahedral coordination, whereas Al^3+^ shows a preference for an octahedral environment.^14,15^ This behaviour is illustrated by the structures of their oxides, where the most thermodynamically stable form of Al_2_O_3_, the α-polymorph, contains solely octahedral Al, whereas for Ga_2_O_3_, the β-polymorph, which contains equal amounts of tetrahedral and octahedral cations, is most thermodynamically stable.^16^ In our recent work on a series of mixed Al–Ga oxides and oxyhydroxides, solid-state ^27^Al and ^71^Ga nuclear magnetic resonance (NMR) spectra confirmed this preference of Ga to occupy tetrahedral sites and Al to occupy octahedral sites.^14^ Mixed Al–Ga spinels provide another example of this site occupancy preference, where short-range cation ordering arising from preferential filling of tetrahedral and octahedral sites was evident.^15^

In this work, we introduce a new strategy to target the synthesis of AlGaPOs using mixed-metal Al–Ga oxides as precursors with the aim of achieving a homogeneous distribution of Al and Ga in the phosphate frameworks, avoiding the phase separation that might conceivably occur if single-metal precursors were to react at different rates. We focused on the chabazite-type framework, AlGaPO-34, since the pure AlPO-34 and GaPO-34 have been reported with a variety of structure directing agents (SDAs), as summarised in Table 1. The as-made structures contain a protonated, hence cationic, form of the SDA along with charge-balancing fluoride, which binds to the framework to give both tetrahedral (MO_4_, M = Al, Ga) and octahedral (MO_4_F_2_) cation sites (denoted M^IV^ and M^VI^, respectively). The question of whether the distribution of Al and Ga over these sites in AlGaPO analogues resembles the distribution of cations in the mixed-metal oxide precursor (a defect spinel, γ-(Al_*x*_Ga_1−*x*_)_2_O_3_) is also an important aspect of this work, with the possibility of the control of cation distribution *via* the reagent used in synthesis. The orientation of the SDA in relation to the phosphate framework, and the amount of water within the pores, can also vary between AlPO and GaPO analogues. This provides further structural complexity for mixed-cation materials.

**Table 1:** Unit cell parameters and volume (space group *P*1̄) of as-made AlPO-34 and GaPO-34 from the literature, synthesised with different SDAs, along with their chemical compositions

Material (SDA)^a^	*a* (Å)	*b* (Å)	*c* (Å)	*α* (°)	*β* (°)	*γ* (°)	*V* (Å^3^)	Ref.	Composition
AlPO-34(pip)	9.3819	9.1644	9.1918	87.760	102.042	93.489	771.20	17	Al_3_P_3_O_12_·F·SDA·0.4H_2_O
AlPO-34(pip)	9.1800	9.1957	9.3606	86.532	78.192	87.739	771.76	18	Al_3_P_3_O_12_·F·SDA·0.25H_2_O
AlPO-34(ipa)	9.1231	9.2411	9.3426	86.769	79.946	87.846	774.0	18	Al_3_P_3_O_12_·F·SDA·H_2_O
AlPO-34(dea)	9.199	9.202	9.295	87.525	79.027	87.884	771.4	18	Al_3_P_3_O_12_·F·SDA·0.5H_2_O
AlPO-34(morph)	9.333	9.183	9.162	88.45	102.57	93.76	764.7	19	Al_3_P_3_O_12_·F·SDA
AlPO-34(pyr)	9.118	9.161	9.335	85.98	77.45	89.01	759.25	20	Al_3_P_3_O_12_·F·SDA·0.15H_2_O
AlPO-34(cyclam)	9.0993	9.2232	9.3929	77.881	87.205	87.777	769.48	21	Al_3_P_3_O_12_·F·SDA
AlPO-34(dmim)^b^	9.0897	9.2075	9.2914	76.546	87.299	89.411	755.45	22	Al_3_P_3_O_12_·F·SDA
GaPO-34(mim)	9.4260	9.1680	9.3080	90.380	103.750	92.580	780.4	23	Ga_3_P_3_O_12_·F·SDA·0.6H_2_O
GaPO-34(pyr)	9.265	9.397	9.238	94.36	90.64	103.67	778.9	24	Ga_3_P_3_O_12_·F·SDA·0.5H_2_O

a pip = piperidine, ipa = isopropylamine, dea = diethylamine, morph = morpholine, pyr = pyridine, cyclam = 1,4,8,11-tetraazacyclotetradecane, dmim = 1,3-dimethylimidazolium, mim = 1-methylimidazolium. Note that all SDAs are in their monocationic (or dicationic for cyclam) forms in the phosphate frameworks.

b Structure determined at 150 K. Estimated standard deviations on lattice parameters are available in the literature ref. 17–24.

Herein we focus on synthesis using the 1-methylimidazolium (mim) SDA. The structure of GaPO-34(mim) has previously been characterised using X-ray crystallography and solid-state NMR spectroscopy^25^ and in the present work we report the first preparation and characterisation of the AlPO end member, AlPO-34(mim), for comparison with the as-made and calcined AlGaPO-34(mim) solid solution. We show that in the as-made AlGaPOs, Al exhibits a strong preference to occupy the octahedral sites, leading to metal ion ordering. In the calcined materials, although ^31^P NMR spectroscopy appears to show a random distribution of cations, we demonstrate that the results are also consistent with the non-random cation distribution seen in the as-made materials.

## Results and discussion

### Structure solution of AlPO-34(mim)

**Fig. 1:**
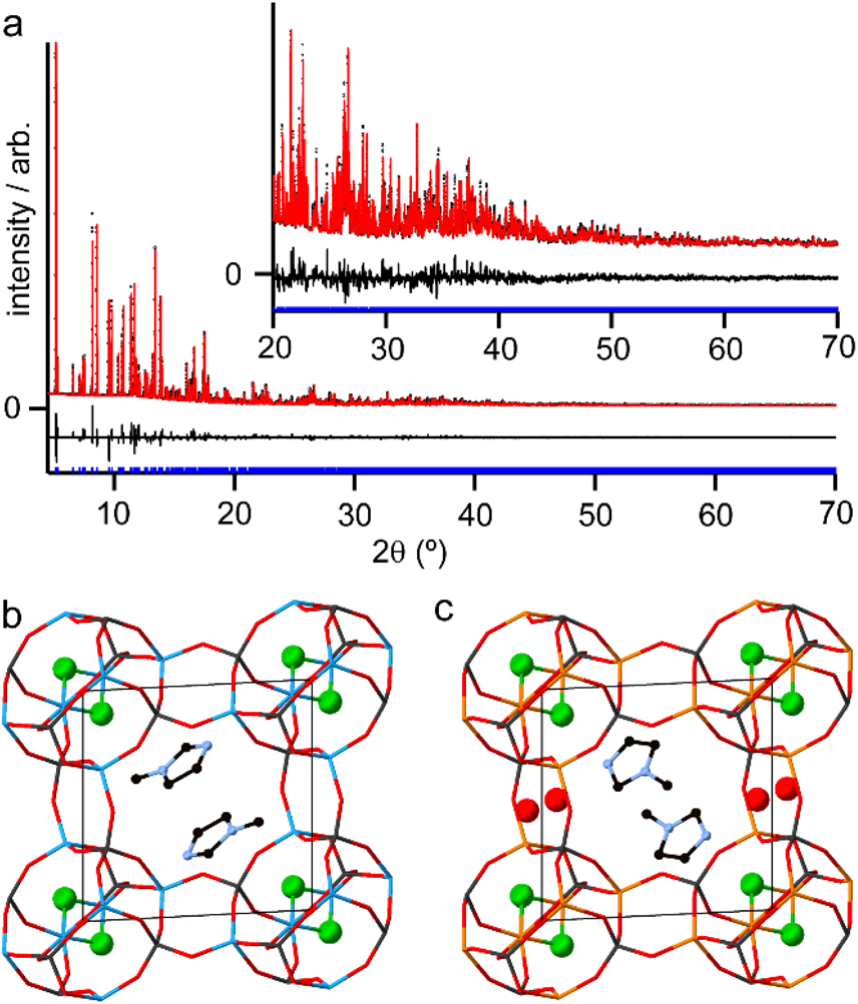
(a) Final Rietveld plot for AlPO-34(mim) with the inset showing an expansion of the region from 20 to 70° 2*θ* with data (black points), fit (red line) and difference curve (black line) and positions of allowed reflections (blue tick marks). Experimentally determined crystal structures of (b) AlPO-34(mim) and (c) GaPO-34(mim) viewed down the crystallographic *c* axes. Atoms are coloured black = C, pale blue = N, red = O, green = F, bright blue = Al, dark grey = P and orange = Ga. The phosphate framework is shown as sticks and other atoms as balls and sticks. H atoms were not located for GaPO-34(mim) and are omitted from the structure of AlPO-34(mim) for clarity.

Although the synthesis of AlPO-34 has been reported with several SDAs already (Table 1), the synthesis and structure solution of AlPO-34(mim) is new to this work. Preparing this material was important to establish to understand the SDA location relative to the inorganic framework and to determine the presence of any occluded water before attempting to understand the structures of the mixed-metal materials. A triclinic unit cell (see Table 2) similar to that of AlPO-34(pip) was found unambiguously (least-squares indexing method) with a satisfactory figure of merit (M_20_ = 319). This led to the suggestion that AlPO-34(pip) and AlPO-34(mim) were isostructural and the atomic coordinates of the inorganic framework of AlPO-34(pip) were then directly used as the starting model in the Rietveld refinement (*P*1̄). The molecules of mim found in the pores were treated as rigid bodies and were then localised using a simulated annealing process in direct space. This decreased the *R*_Bragg_ value from 0.29 to 0.17. The structural model was then refined using the Rietveld method, which at the final stage involved the following structural parameters: 57 atomic coordinates of the inorganic framework, 6 parameters for the position and orientation of the mim as well as 3 distances and the torsion angle of its methyl group, 4 thermal factors, and 1 scale factor for 4331 reflections. The final Rietveld plot (Fig. 1a) corresponds to satisfactory model indicator (*R*_Bragg_ = 0.046) and profile factors (*R*_p_ = 0.043 and *R*_wp_ = 0.064). Difference Fourier map calculations did not show any residual density that could correspond to a water molecule. Fig. 1b shows the final structure, where it is compared with the structure of GaPO-34(mim).^23^ Although the phosphate frameworks are isostructural a difference in orientation of the SDA between the two structures can be seen (discussed further below), and the GaPO-34(mim) also includes extraframework water.

**Table 2:** Selected crystallographic parameters for AlPO-34(mim)

Empirical formula	Al_3_ P_3_ F O_12_ C_4_N_2_H_7_
*M*_r_ (g mol^−1^)	467.97
Crystal system	Triclinic
Space group	*P*1̄
*a* (Å)	9.30794(7)
*b* (Å)	9.15665(7)
*c* (Å)	9.15459(9)
*α* (°)	88.9517(7)
*β* (°)	102.1483(7)
*γ* (°)	93.1108(7)
*V* (Å^3^)	761.63(1)
*Z*	2
*λ* (Å)	0.826855
Number of reflections	4331
Number of fitted structural parameters	72
Number of soft restraints	3
*R*_p_, *R*_wp_	0.043, 0.064
*R*_Bragg_, *GoF*	0.046, 5.66

**Fig. 2:**
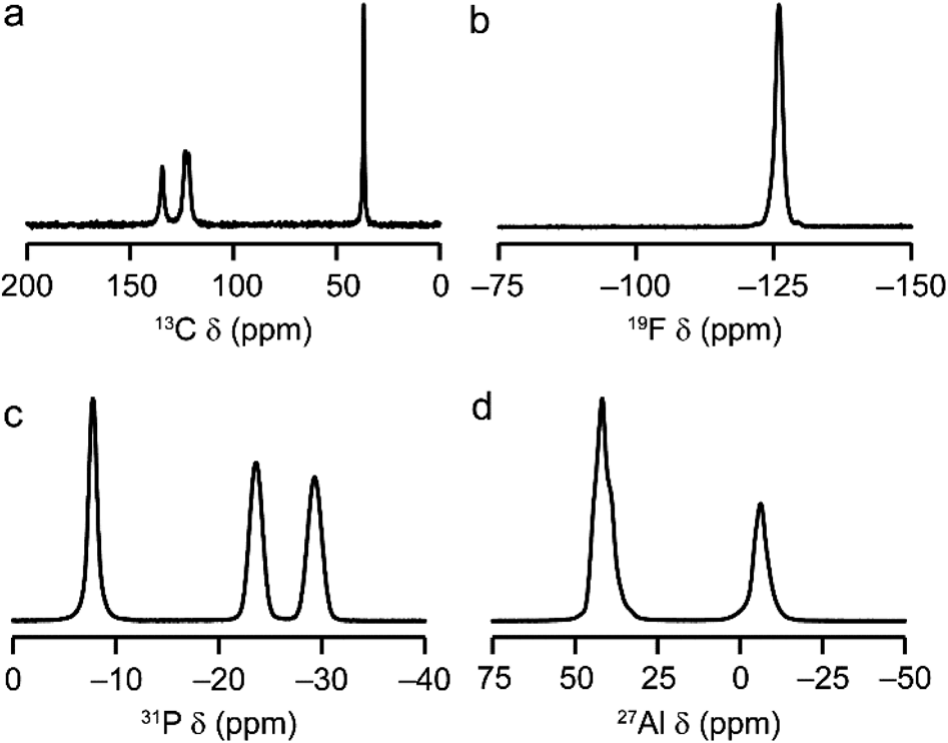
Solid-state NMR spectra of as-made AlPO-34(mim): (a) ^13^C (9.4 T, 12.5 kHz CP MAS), (b) ^19^F (14.1 T, 50 kHz MAS), (c) ^31^P (9.4 T, 14 kHz MAS), (d) ^27^Al (9.4 T, 14 kHz MAS). Further experimental details are given in Table S1.†

AlPO-34(mim) was also characterised by solid-state NMR spectroscopy. The spectra, shown in Fig. 2, are consistent with our earlier work on AlPO-34 prepared with six different SDAs.^17^ The ^13^C CP MAS NMR spectrum (Fig. 2a) contains four distinct resonances at 134.5, 123.5, 121.7 and 36.9 ppm, in agreement with the crystal structure, in which the two mim within a pore are related by an inversion centre. The ^19^F NMR spectrum (Fig. 2b) contains a resonance at −126.0 ppm, consistent with the shifts for bridging Al–F–Al species seen previously in AlPO-34.^17^ The ^31^P MAS NMR spectrum (Fig. 2c) contains three signals at −7.8, −23.6 and −29.3 ppm, which can be assigned respectively to P1, P2 and P3 by comparison to density functional theory (DFT) calculations. The ^27^Al MAS NMR spectrum (Fig. 2d) contains two signals corresponding to tetrahedral and octahedral Al in a 2 : 1 integrated intensity ratio, and the signals for the two tetrahedral Al sites can be separated using a triple-quantum (3Q) MAS experiment (shown in Fig. S3†). The three ^27^Al signals have isotropic chemical shifts of −3.4, 45.5 and 46.8 ppm, and can be assigned to octahedral (Al1) and tetrahedral (Al2 and Al3) sites, respectively, again by comparison with DFT calculations. The experimental and calculated ^31^P and ^27^Al NMR parameters are compared in Table 3 and, while the ^31^P chemical shifts are well reproduced by calculation, the ^27^Al NMR parameters, particularly for the octahedral Al1 site, are poorly reproduced. This may indicate a difference between the room temperature NMR experiments and the effectively 0 K static (optimised) crystal structure used for the calculations. Indeed, as observed previously for other forms of AlPO-34,^17^ there is likely to be microsecond timescale dynamics in AlPO-34(mim), which affect the ^27^Al MAS NMR spectra, although that is not the focus of the present study.

**Table 3:** Experimental (exp.) and calculated (calc.) ^31^P and ^27^Al NMR parameters of AlPO-34(mim)

Site	*δ*_iso_ (ppm)		*C*_Q_ (MHz)		*η*_Q_	
	exp.	calc.	exp.	calc.	exp.	calc.
P1	−7.8(1)	−8.6				
P2	−29.3(1)	−28.8				
P3	−23.6(1)	−22.7				
Al1	−3.4(5)	7.1	2.1(2)	0.9	0.5(1)	0.29
Al2	45.5(5)	46.1	2.3(1)	1.9	0.69(5)	0.83
Al3	46.8(5)	48.1	3.1(1)	2.6	0.46(5)	0.34

### Synthesis of AlGaPOs

The powder X-ray diffraction (PXRD) patterns shown in Fig. S4† confirm the synthesis of phase-pure AlGaPO-34(mim) materials with general formula (Al_*x*_Ga_1−*x*_)_3_P_3_O_12_·F·mim·*y*H_2_O with nominal *x* values of 0.25, 0.5 and 0.75. The actual value of *x* in the materials was verified by ICP-MS analysis and was found to be in good agreement with the expected values from the parent oxides. The water content, *y*, was determined using thermogravimetry (Fig. S5†) and varied from 0.15 (*x* = 0) to 0.68 (*x* = 0.75), as shown in Table S2.† In our previous work on as-made AlPOs, including AlPO-34, the water content of the materials was shown to depend on factors including the initial drying of the powder following synthesis and the presence of moisture in the air during storage, with some frameworks able to absorb ambient moisture on a timescale of days to weeks.^17,26,27^ As such, it is not surprising that there is no clear relationship between water content and Al/Ga ratio. The possible structural significance of the water content of the AlGaPOs is discussed in further detail below.

The PXRD patterns of the AlGaPOs (Fig. S4†) were fitted to obtain lattice parameters, but this revealed no clear trends. Disorder, in the form of variable water content, mixed occupancy of the metal sites and variable orientation of the SDA (see below) leads to broadening of the patterns such that refinement of the Al and Ga occupancies of the three metal sites was not possible using the laboratory PXRD data acquired here.

### Solid-state NMR spectroscopy of AlGaPOs

**Fig. 3:**
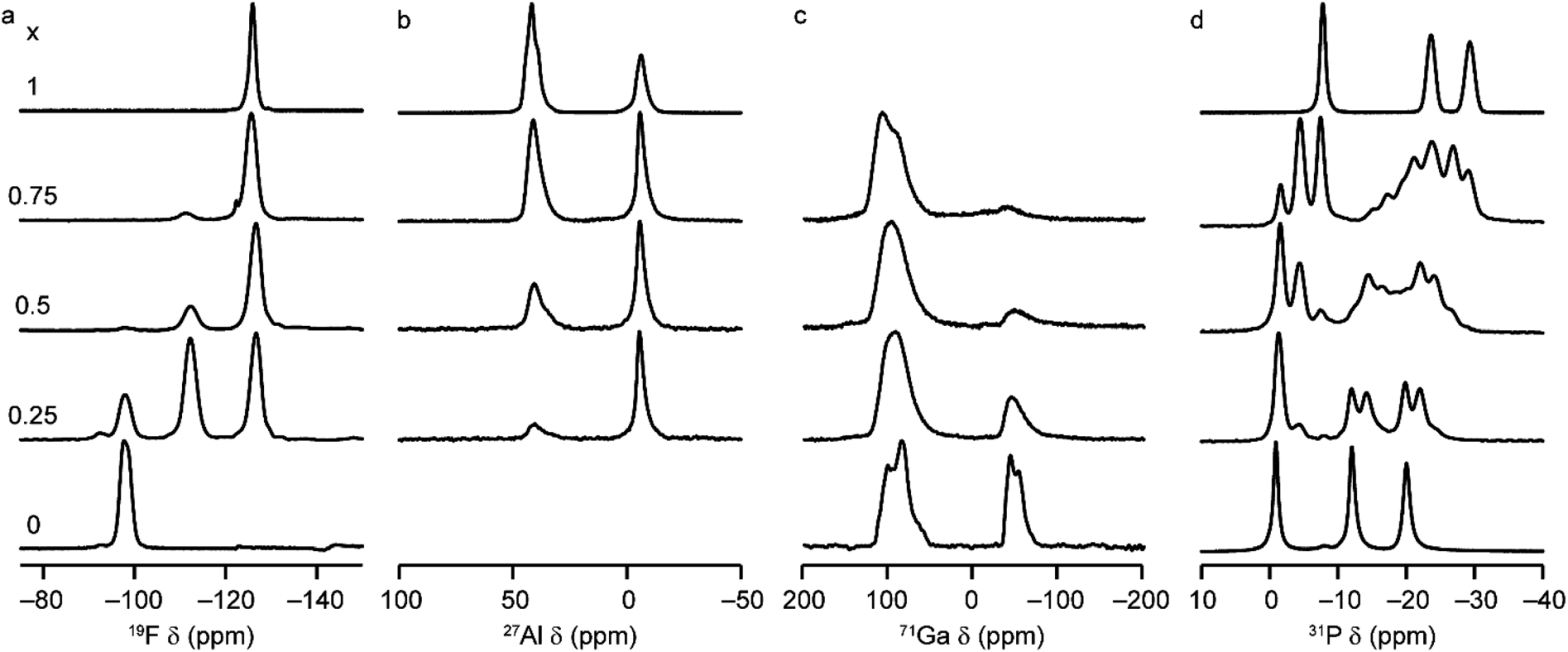
(a) ^19^F (14.1 T, 25–40 kHz MAS), (b) ^27^Al (9.4 T, 14 kHz MAS), (c) ^71^Ga (20.0 T, 55 kHz MAS) and (d) ^31^P (14.1 T, 14 kHz MAS) NMR spectra of as-made (Al_*x*_Ga_1−*x*_)_3_P_3_O_12_·F·mim with *x* = 0, 0.25, 0.5, 0.75 and 1.0.

Solid-state NMR spectroscopy was used to gain a detailed insight into the local arrangement of the Al and Ga cations within the materials. Fig. 3 shows the ^19^F, ^27^Al, ^71^Ga and ^31^P MAS NMR spectra of the five (Al_*x*_Ga_1−*x*_)_3_P_3_O_12_·F·mim samples. The ^19^F NMR spectra of the AlPO and GaPO end members contain resonances at −126.0 and −98.0 ppm, respectively, consistent with the literature.^17,25^ Both signals are present with varying intensity in the mixed-metal materials, indicating the presence of Al–F–Al and Ga–F–Ga linkages. However, there is a third resonance, at around −112 ppm (exactly midway between the other two signals), which indicates the presence of Al–F–Ga linkages and confirms that Al and Ga are mixed on the atomic level. Schott-Darie *et al.*^12^ observed a similar effect in mixed-metal Al/Ga cloverite frameworks, although the fluoride in those materials is contained in a *d4r* cage with four relatively long bonds to the metal cations (rather than two relatively short bonds as is the case in the AlGaPO-34 materials), such that the shift difference for each addition of Al (and loss of Ga) in the coordination environment is around −7.2 ppm for cloverite as opposed to the −14 ppm shift difference observed here.

The ^27^Al MAS NMR spectra contain signals for Al^IV^ and Al^VI^ and, likewise, the ^71^Ga NMR spectra contain signals for Ga^IV^ and Ga^VI^ (note that a much higher field of 20.0 T is required to obtain baseline separation for ^71^Ga signals owing to the larger second-order quadrupolar broadening). The as-made forms of AlPO-34 and GaPO-34 contain two crystallographically distinct tetrahedral Al/Ga sites, but these cannot be resolved in the MAS spectra. ^27^Al 3Q MAS experiments were carried out to resolve the signals from the two Al^IV^ sites as shown in Fig. S6.†

**Fig. 4:**
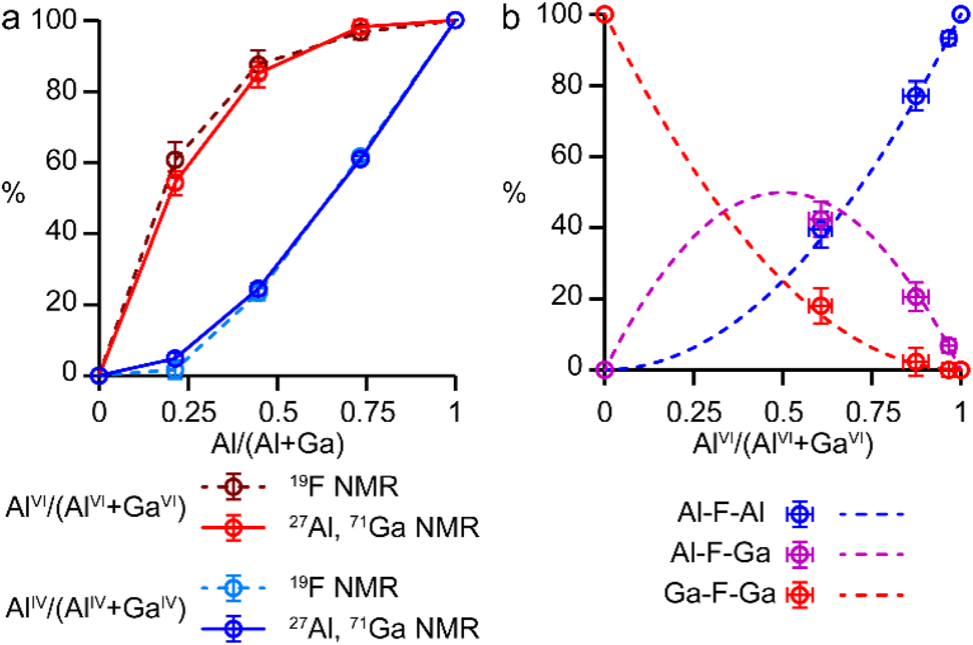
(a) Plots of the fraction of octahedral sites occupied by Al, Al^VI^/(Al^VI^ + Ga^VI^), in red, and of tetrahedral sites occupied by Al, Al^IV^/(Al^IV^ + Ga^IV^), in blue, as a function of composition for (Al_*x*_Ga_1−*x*_)_3_P_3_O_12_·F·mim, determined from a combination of ^27^Al and ^71^Ga NMR spectroscopy (solid lines) and from ^19^F NMR spectroscopy (dashed lines). (b) Plots of expected relative intensities of the Al–F–Al, Al–F–Ga and Ga–F–Ga signals against octahedral site occupancy, assuming a random distribution of the cations (dashed lines) and their experimental intensities (circles). In both parts error bars indicate estimated uncertainties in the ^71^Ga and ^19^F spectral integrals.

It is possible to gain insight into the cation distributions within (Al_*x*_Ga_1−*x*_)_3_P_3_O_12_·F·mim by combining information from the ^19^F, ^27^Al and ^71^Ga MAS NMR spectra. The ^27^Al NMR spectra provide information on the fraction of the Al that is on a tetrahedral or octahedral site(s) and the ^71^Ga NMR spectra contain information on the corresponding fractions of tetrahedral and octahedral Ga. By combining this information with the composition of the materials (determined using ICP-MS), it is possible to determine the Al content on the octahedral and tetrahedral sites. This is plotted in Fig. 4 (solid lines) and shows that the octahedral sites are enriched in Al relative to the tetrahedral sites in the mixed-metal materials. This observation is consistent with earlier literature on mixed-metal Al/Ga oxides and oxyhydroxides, and spinels in which octahedral sites were enriched in Al.^14,15^ While the ^27^Al 3QMAS spectra (shown in Fig. S6†) are not quantitative, the relative intensities of the two Al^IV^ signals do not change significantly with varying Al content, indicating that there is no particular preference for Al or Ga to occupy one or the other of the tetrahedral sites.

The ^19^F NMR spectra contain information regarding the composition of the octahedral sites. Again, by combining this with the composition of the materials, it is possible to arrive at an independent measure of the Al content on the octahedral and tetrahedral sites (dashed lines in Fig. 4a). These values are in excellent agreement with those determined directly from the ^27^Al and ^71^Ga NMR spectra.

It is clear from the data presented in Fig. 3 and 4 that the Al and Ga are not randomly distributed over the octahedral and tetrahedral sites, with Al exhibiting a strong preference for octahedral coordination and Ga a strong preference for tetrahedral coordination. However, there is an additional preference that is relevant to consider for the AlGaPO-34 structure. The fluoride anions bridge pairs of octahedral cations, forming Al–F–Al, Al–F–Ga, or Ga–F–Ga linkages, and it is possible that one of these will be favoured or disfavoured over the others. Fig. 4b plots the relative proportions of these three linkages (determined from integrating the signals in the ^19^F NMR spectra). When compared to the proportions expected using a random distribution (dashed lines in Fig. 4b), around 5% fewer Al–F-Ga linkages are observed than would be expected for the sample with the least Al, although this is within the error of the experimental measurement. Therefore, there is a strong preference for Al to occupy octahedral sites, but there does not appear to be a significant preference for pairs of Al (or pairs of Ga) to occupy adjacent octahedral sites. These results confirm the homogeneity of mixing of the two metal cations within the AlGaPOs, with no evidence of clustering on the nanoscale.

**Fig. 5:**
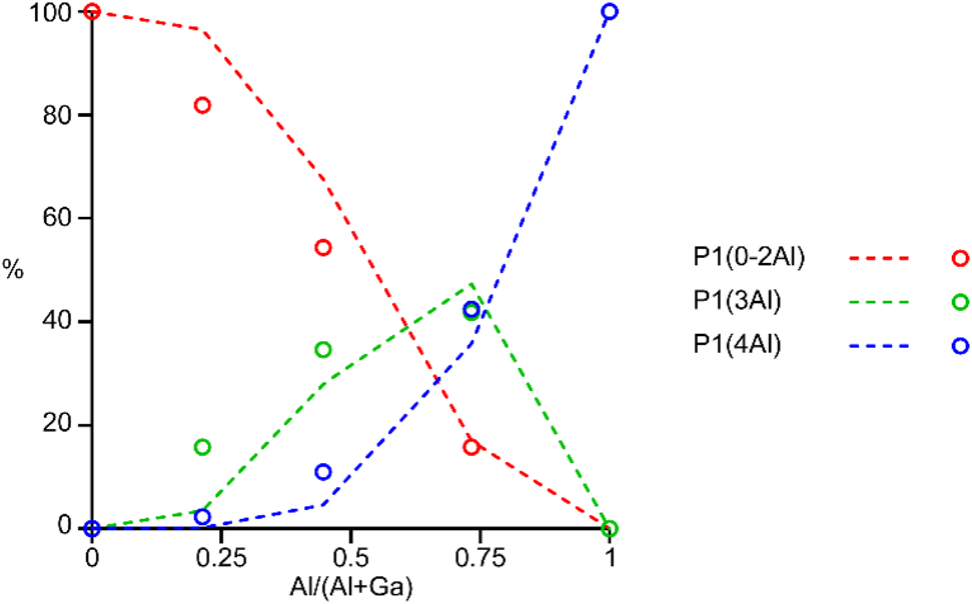
Plot of the experimental intensities (circles) of the three P1 signals as a function of *x* in (Al_*x*_Ga_1−*x*_)_3_P_3_O_12_·F·mim compared to those predicted by the model described in the ESI† (dashed lines) assuming that signals result from P(0-2Al), P(3Al) and P(4Al).

The ^31^P MAS NMR spectra of the mixed-metal samples (shown in Fig. 3), are complex, with the signals for each of the three crystallographic P sites split into multiple resonances, corresponding to different numbers of P–O–Al and P–O–Ga linkages. This splitting is analogous to the well-known splitting of ^29^Si signals in aluminosilicates.^28^ For P2 and P3 (using the site labelling of Dawson *et al.*^17^), there is significant overlap of the complex lineshapes for each site, making detailed interpretation very challenging. However, the signals for P1 occur at higher shift and can be investigated in more detail. The first point to note is that, although one might (as in zeolite frameworks) expect five signals for P(OAl)_*n*_(OGa)_4−*n*_ (0 ≤ *n* ≤ 4), only three signals are observed for P1 in the mixed-metal materials, at −1.3 to −1.6 ppm, −4.4 ppm, and −7.4 to −7.9 ppm. This compares to the shifts for the AlPO and GaPO end members of −0.9 and −7.8 ppm, respectively, and leads naturally to the question of the assignments of these three signals. As discussed in further detail in the ESI,† a model in which the P(0Al), P(1Al) and P(2Al) signals are overlapped and contribute to the signal with the highest shift, whereas the P(3Al) and P(4Al) signals appear separately and contribute to the signals at −4.4 and −7.4 to −7.9 ppm, respectively, gives reasonable agreement with the experimentally observed intensities. The results of using this model are shown in Fig. 5.

DFT calculations were carried out to support and rationalise some of the findings from the NMR spectra. The structural models from XRD show that the SDA is in different orientations for the two end members (see Fig. 1b and c), and the water content of the AlGaPOs varies between samples, making the generation of structural models of AlGaPOs for DFT calculations nontrivial. As shown in Fig. S7,† small shift differences in the ^13^C CP MAS NMR spectra of the materials support this variation in SDA orientation. Additionally, it is likely that the water and SDA will be dynamic on the microsecond timescale, as observed in a range of AlPO-34 frameworks.^17^ These factors were partially mitigated by carrying out two series of calculations with the SDA in the orientation seen for each end member, with no water present. This choice of structural models allows us to investigate general trends for Al/Ga substitution in fully anhydrous materials. A smaller set of additional structural models with water molecules in locations corresponding to that observed experimentally for GaPO-34 (ref. 23) were used to investigate the structural and energetic effects of hydration. These are discussed further in the ESI.†

**Fig. 6:**
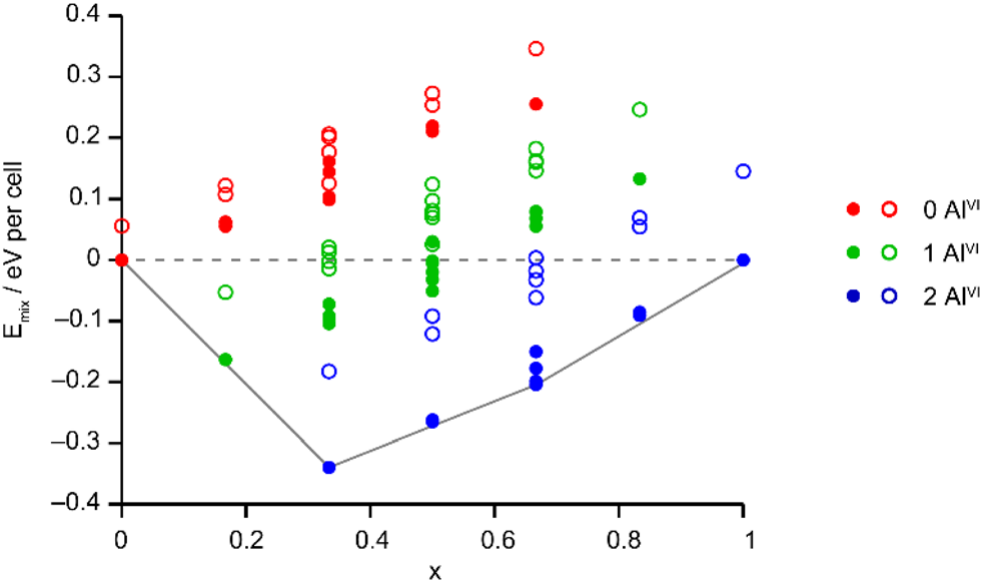
Plot of *E*_mix_ against *x* for the 72 distinct arrangements of Al and Ga in the AlGaPO-34(mim) structural models with the SDA in the orientation matching the AlPO end member (filled circles) or anhydrous GaPO end member (open circles). The dashed grey line indicates *E*_mix_ = 0 and the solid grey line is the convex hull.

The Site Occupancy Disorder (SOD) program^29^ was used to generate starting models containing all symmetry-distinct arrangements of Al and Ga on the metal sites. The models were optimised to minimise atomic forces, and the energy and NMR parameters were calculated for each optimised structure. The mixing enthalpies (where *E*_mix_ = *E*_(AlGaPO)_ − *xE*_(AlPO)_ − (1 − *x*)*E*_(GaPO)_ and *x* is from the composition of the AlGaPO) of the models are plotted in Fig. 6 for models based on the AlPO-34(mim) structure (filled points) and on the GaPO-34(mim) structure (open points). For the anhydrous structural models, those based on the AlPO end member are slightly more stable (by around 0.05–0.15 eV), but those based on the GaPO end member follow a similar pattern. At each composition there are models with negative mixing enthalpy, *i.e.*, mixing is exothermic and the series is expected to display solid solution behaviour. While this approach considers only enthalpy, the mixing entropy will always be positive, such that some structures above the *E*_mix_ = 0 line may also have negative Gibbs free energy. The convex hull (solid grey line in Fig. 6) is asymmetric, with a minimum at *x* = 1/3 (*i.e.*, two Al per unit cell). Inspection of the structures lying on (or close to) the convex hull shows that in all cases, the most stable structures maximise the number of Al^VI^ present, with each Al^VI^ providing around 0.2 eV (around 20 kJ mol^−1^) stabilisation. This stabilisation is almost an order of magnitude greater than that calculated in earlier work for mixed Al/Ga oxides and oxyhydroxides,^14^ where the stabilisation from having Al^VI^ + Ga^IV^, rather than *vice versa*, was between 2 and 5.2 kJ mol^−1^. However, in the oxide-based materials studied earlier, the octahedral sites were MO_6_ or MO_5_OH, whereas in the present phosphate materials, the octahedral sites are MO_4_F_2_, suggesting that in these phosphate frameworks the Ga–F bonds are significantly weaker than Al–F bonds, while Ga–O bonds are only slightly weaker than Al–O bonds. This is consistent with the lower dehydrofluorination temperature observed for GaPO-34 (ref. 30) compared with AlPO-34.^17^ Ga^3+^ is also softer (has a greater tendency to form more covalent bonds) than Al^3+^, so is likely to favour the smaller tetrahedral site rather than the larger but higher-coordinate octahedral site.

**Fig. 7:**
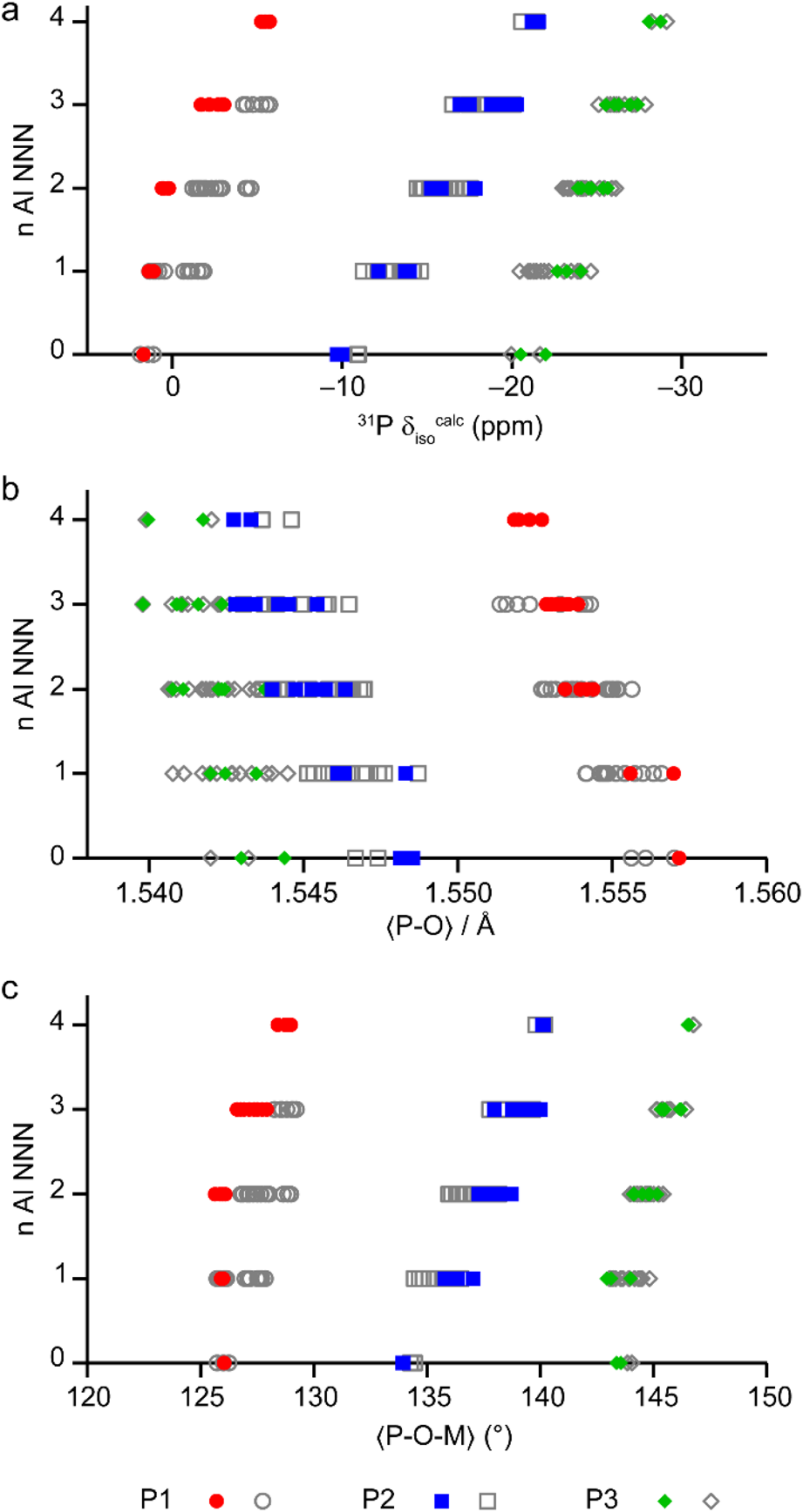
(a) Calculated ^31^P *δ*_iso_ for P sites with 0–4 Al NNN in the 36 distinct structural models for as-made AlGaPO-34 with the SDA in the orientation matching the AlPO end member, and with no water present. (b and c) Plots of the mean P–O bond length (b) and mean P–O–M (M = Al, Ga) bond angle (c), separated by number of Al NNN in the same 36 structural models. Points on or near the convex hull (see Fig. 6) are shown in colour, whereas the less energetically favorable structures are shown in grey.

The calculated NMR parameters can be used to confirm the assignment of the ^19^F NMR spectra (see the ESI†) and can also be used to gain further understanding of the ^31^P NMR spectra. Fig. 7 shows the calculated ^31^P *δ*_iso_ for all models in the series based on the AlPO parent structure, separated by the number of next-nearest neighbour (NNN) Al. For P1 (circles) there is significant overlap between the ranges of chemical shifts predicted for P(*n*Al) and P((*n* ± 1)Al). However, when only the shifts from the lowest energy structures (*i.e.*, those on or close to the convex hull) are considered for P1 (red points in Fig. 7) the calculated shifts for P(0–2Al) are coincident while P(3Al) and P(4Al) are separated. This result confirms both the assignment of the experimental signals as discussed above and that the AlGaPO-34 frameworks adopt predominantly the minimum energy configuration(s) for the metal cations. Interestingly, for P2 and P3, the ^31^P *δ*_iso_ is still predicted to vary systematically with *n*, although the ranges for P2(*n*Al) and P3(*n*Al) would be expected to overlap, as observed experimentally. Note that while the calculated *δ*_iso_ values shown in Fig. 7 reproduce experimental trends well, the precise values are a poor match to experiment. This is most likely because the set of model structures shown do not include the water molecules that were observed by thermogravimetric analysis (TGA, see Table S2†).

The question remains, however, as to why the ^31^P chemical shift for P1 cannot distinguish between zero and two Al NNN but can then clearly distinguish between three and four Al NNN, whereas for P2 and P3 in the same materials, the ^31^P chemical shifts are systematically sensitive to the number of Al NNN. Previous work on AlPOs has shown that the ^31^P *δ*_iso_ depends strongly on both the mean P–O bond length and the mean P–O–Al bond angle,^7,31–33^ but in optimised model structures (*i.e.*, those representing local minima for a particular arrangement of atoms), the P–O bond lengths tend to be very similar. As shown in Fig. 7b and c, when considered over the whole series of model structures for the solid solution, both the mean P–O bond length and the mean P–O–M bond angles vary systematically with the number of NNN Al for all three P sites. However, when only the low energy structures for each composition are considered, the mean P–O–M angle for P1 is consistent at 126.0(1)° for 0, 1 or 2 NNN Al, but then varies significantly (to 127.0° and 128.8° for 3 and 4 NNN Al, respectively). It is interesting to note that, although the energetics of the system are governed by the number of octahedral Al, the variation in the local structure around the P sites (and, hence, the ^31^P *δ*_iso_) is mainly governed by changes to the geometry of the P–O–M linkages with the tetrahedral cations.

As discussed above, the ^19^F MAS NMR spectra (Fig. 3a and 4b) gave limited evidence of any preference for the formation of Al–F–Al and Ga–F–Ga linkages rather than mixed-metal Al–F–Ga linkages. This preference was investigated computationally using a 2 × 1 × 1 supercell containing two pairs of octahedral cations. When two Al were placed in the GaPO framework, a very small preference (around 0.01 eV, 1.1 kJ mol^−1^) for Al–Al and Ga–Ga pairs was observed, whereas when two Ga were placed within the AlPO framework, no preference was observed. This suggests that any preference for like pairs of octahedral cations is negligible, which is consistent with the experimental results discussed above.

**Fig. 8:**
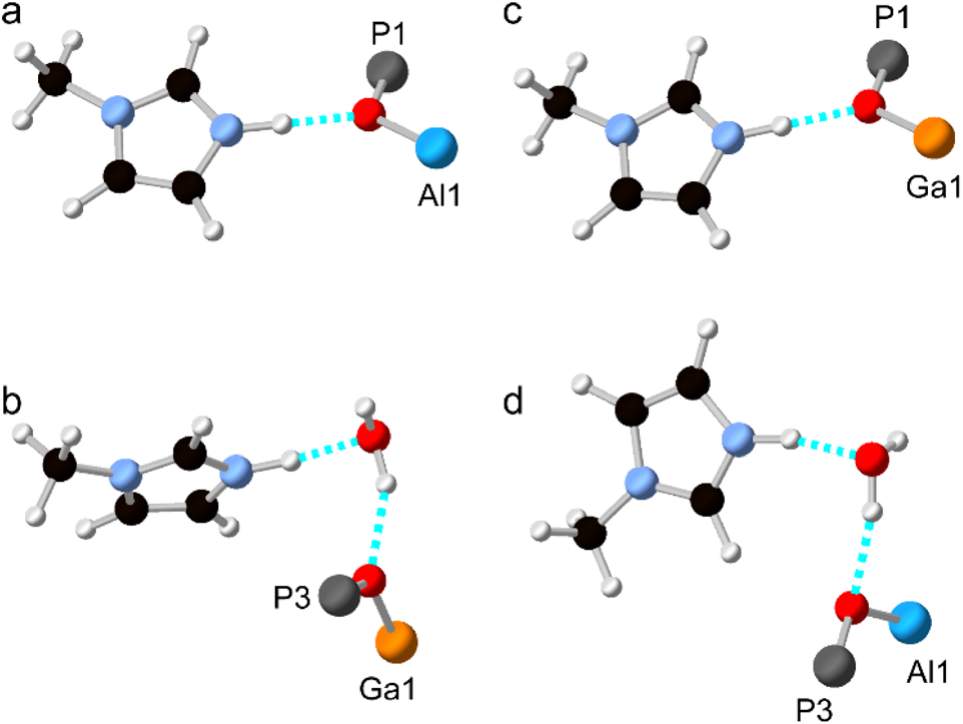
Hydrogen bonds formed by the SDA in DFT-optimised models of anhydrous and hydrated AlPO-34(mim) and GaPO-34(mim). (a) The optimised experimental structure of (anhydrous) AlPO-34(mim), (b) the optimised experimental structure of (hydrated) GaPO-34(mim), (c) “anhydrous” GaPO-34(mim) made by replacing Al with Ga in the experimental structure from (a), and (d) “hydrated” AlPO-34(mim) made by replacing Ga with Al in the experimental structure from (b). Atoms are coloured white = H, black = C, pale blue = N, red = O, bright blue = Al, dark grey = P and orange = Ga. Hydrogen bonds are indicated in dashed cyan.

As noted above, and shown in Fig. 1, the SDA has a markedly different orientation in as-made AlPO-34(mim) and GaPO-34(mim). As shown in Fig. 8a, in the DFT-optimised experimental structure of AlPO-34(mim), the SDA can form a N–H⋯O hydrogen bond with the framework oxygen atom bridging P1 and Al1. The N–H bond length is 1.050 Å, the H⋯O distance is 1.717 Å and the N–H–O angle is 164.4°. In addition to the SDA, the crystal structure of the as-made GaPO contains a partially occupied water site, which, when occupied, may act as a hydrogen bond acceptor and influence the orientation of the SDA. As seen in Fig. 8b, for the DFT-optimised structure of GaPO-34(mim),when the water molecule is present, the SDA forms a N–H⋯O hydrogen bond with the water, and the water forms a O–H⋯O hydrogen bond to the oxygen between P3 and Ga1. However, as there is only one molecule of water in the structural model, the other mim cannot form an equivalent hydrogen bond to water and instead exists as the “free” cation within the pore rather than forming any hydrogen bonds (the shortest H⋯X contact is 2.242 Å to a framework oxygen and the N–H bond length is 1.024 Å). This difference in the SDA behaviour may arise partly from the presence of the water, but also the higher ionic character of the AlPO_4_ framework relative to the more covalent GaPO_4_ framework. This difference can be explained qualitatively by the relative Sanderson electronegativities of Al, Ga and P, of 1.71, 2.41 and 2.51, respectively (or 1.61, 1.81 and 2.19, respectively on the Pauling scale).^13,34^ As shown in the ESI,† the Mulliken charges calculated by CASTEP for the framework O atoms in the AlGaPOs support this explanation with Al–O–P being around 0.1 |*e*| more negative than Ga–O–P. Consequently, the more ionic AlPO framework may act as a better hydrogen-bond acceptor and exert more influence over the orientation/reorientation of the SDA than the GaPO framework. In the as-made mixed-metal AlGaPOs, from one unit cell to the next, the SDA in the “same” orientation may encounter Al–O–P, Ga–O–P or H_2_O as the hydrogen bond acceptors, leading to potentially very complicated average behaviour.

Structural models were also considered where all of the Al atoms in the experimental AlPO structure were replaced by Ga, and *vice versa*. The hydrogen bonding in these structural models (after optimisation) is shown in Fig. 8c and d and the geometry of the hydrogen bond is similar whether the metal cations are Al or Ga. The geometries of the hydrogen bonds are summarised in Table 4. However, there are subtle differences between the two hydrated structures (Fig. 8b and d), where, for the AlPO, the water molecule is rotated by around 150° about the O–H⋯O “axis” and the mim is rotated by around 90° about the N–H⋯O “axis” relative to the GaPO (although since all atomic coordinates and the unit cell parameters are different, these rotation angles are, by necessity, only approximate). Interestingly, when the water molecule is removed from the experimental structure of the GaPO, and the model optimised by DFT, the SDA does not reorient to form hydrogen bonds with framework cations, instead remaining as a “free” cation with a N–H bond length of 1.025 Å. This model is 2.7 kJ mol^−1^ more stable than the “dehydrated GaPO” model structure, which indicates both that the DFT calculations may not have explored enough of the conformational and orientational space of the SDA to locate the true global minimum, and that there are probably multiple thermally-accessible local minima for the SDA. In other words, the SDA may well be dynamic at room temperature and the orientation of any given molecule will likely also be influenced by any water molecules present. However, a more comprehensive computational exploration of the potential energy surface for the SDA and any water present is beyond the scope of this work and appears unnecessary to understanding the cation distribution within the framework. The likely very complicated disorder of the pore contents of as-made AlGaPO-34 may explain the appearance of both the broadened reflections in the PXRD patterns and the non-systematic variation in chemical shifts in the ^13^C CP MAS NMR (see Fig. S4 and S7†).

**Table 4:** Optimised covalent and hydrogen bond lengths and bond angles in the four structural models shown in Fig. 8. Data from the experimental (exp) structure of the AlPO with the N–H distance fixed at 1.00 Å is included for comparison

Structure		X–H (Å)	H⋯O (Å)	Angle (°)
AlPO (exp)	N–H⋯O	1.00	1.875(8)	138.2(6)
AlPO	N–H⋯O	1.05	1.72	164.4
GaPO	N–H⋯O	1.05	1.73	162.4
GaPO + H_2_O	N–H⋯O	1.06	1.68	168.7
	O–H⋯O	0.98	1.95	156.2
AlPO + H_2_O	N–H⋯O	1.06	1.67	166.4
	O–H⋯O	0.98	1.98	160.0

Given the probable SDA dynamics and the fact that N and C are both light atoms with similar X-ray scattering factors, we also considered a series of structural models with the N atom on the “4” position of the ring rather than the “3” position (*i.e.*, corresponding to a 180° rotation of mim about the H_3_C–N bond). The models are described in further detail in the ESI† but, in general, the trends in their energies were similar to those shown in Fig. 8. The experimental anhydrous AlPO-34(mim) structure (Fig. 8a) was determined to be around 25.6 kJ mol^−1^ more stable than the model with the N in position 4 of the ring. In the modified structure, the mim does not form a hydrogen bond and, instead, the parts of the SDA closest to the framework are the CH now on position 3 and one of the methyl H. These results further support the hypothesis that there is likely to be a rather flat potential energy surface for the SDA orientation and this will be affected by multiple factors, including hydration state and the Al/Ga content and (dis)ordering of the framework as well as the ability to form SDA⋯framework hydrogen bonds. Additionally, there is good agreement between the DFT calculations and the structural models suggested by PXRD in the position of the N atoms in the SDA for both experimental structures (anhydrous AlPO and hydrated GaPO).

### Characterisation of calcined AlGaPOs

**Fig. 9:**
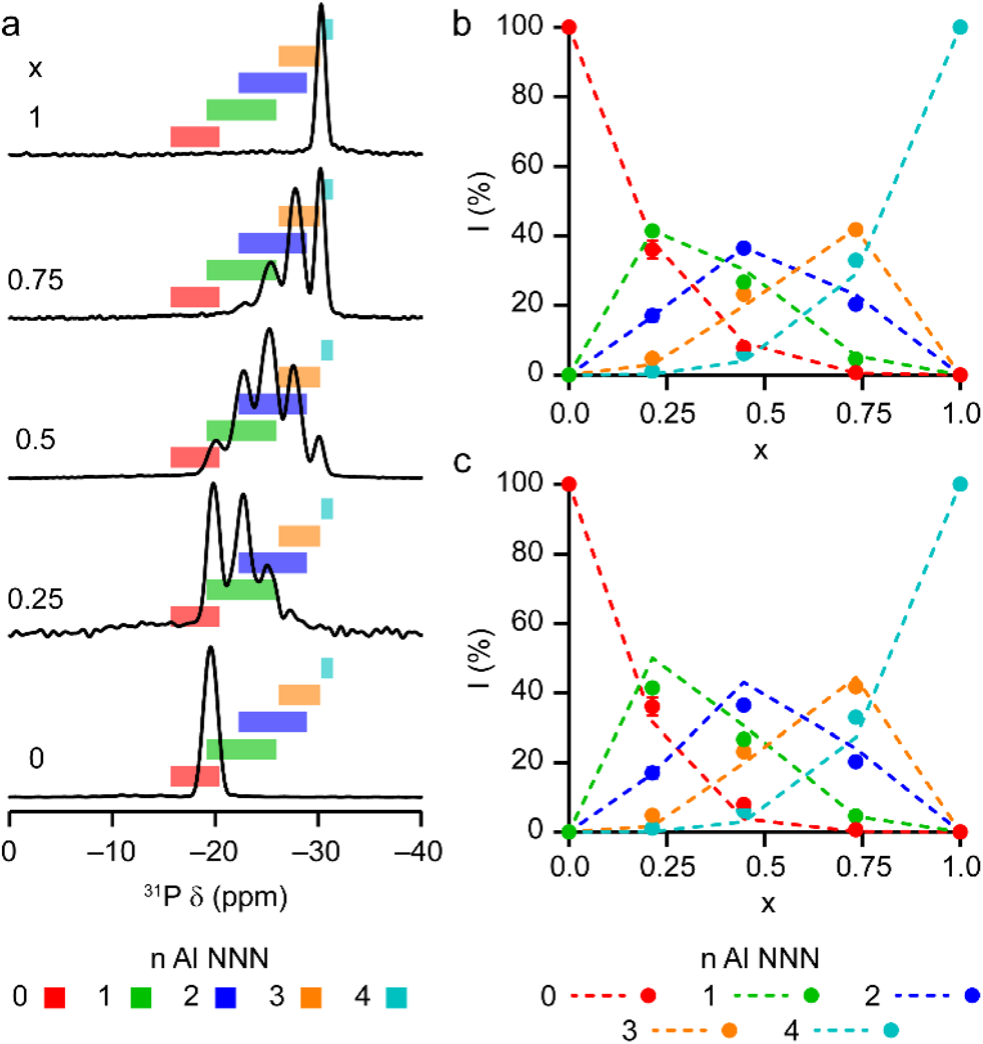
(a) ^31^P (20.0 T, 50 kHz MAS) NMR spectra of calcined dehydrated Al_*x*_Ga_1−*x*_PO_4_-34 with *x* = 0.25, 0.5, 0.75 and 1.0. The ^31^P (14.1 T, 25 kHz MAS) NMR spectrum of calcined dehydrated GaPO-34 (ref. 24) is also shown. Coloured boxes indicate the ranges of ^31^P *δ*_iso_ calculated for the 14 distinct arrangements of Al and Ga in calcined AlGaPO-34, separated by number of Al NNN. (b and c) Plots of experimental ^31^P peak intensities compared with the intensities predicted for (b) a random distribution of Al and Ga cations and (c) a distribution of Al and Ga cations based on the occupancy of the octahedral and tetrahedral sites of the as-made materials. See the ESI† for further detail of the two models.

**Fig. 10:**
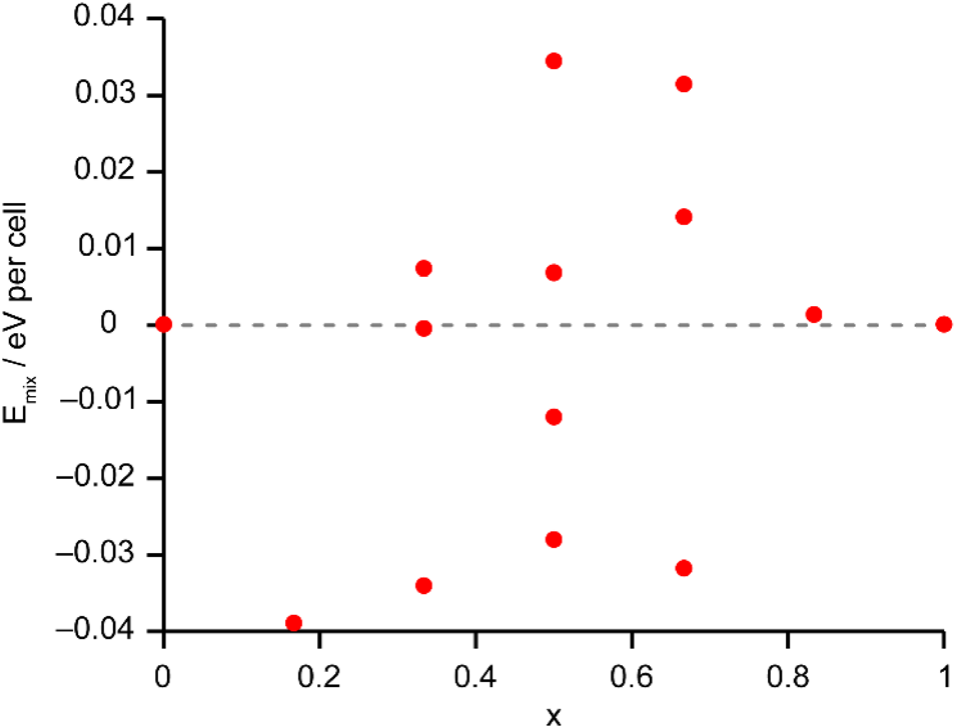
Plot of *E*_mix_ against *x* for the 14 distinct arrangements of Al and Ga in the calcined AlGaPO-34 structural models. Note that the energy scale covers 10% of that shown in Fig. 6 for the as-made materials. The dashed grey line indicates *E*_mix_ = 0.

The solid-state ^27^Al and ^71^Ga MAS NMR spectra of the calcined, dehydrated AlGaPOs (see Fig. S17†) confirm that the metal cations are all in a tetrahedral M(OP)_4_ environment, leading to a single sharp signal in both sets of spectra. The ^31^P MAS NMR spectra of the calcined dehydrated AlGaPOs (shown in Fig. 9a) are also simplified relative to the as-made materials, with a single crystallographic P site. For the AlGaPOs, the resonance is split into five distinct contributions, evenly spaced between the shifts of the AlPO_4_ end member (−30.3 ppm) and the GaPO end member (−19.6 ppm). By comparison with the DFT calculations, the signals in the ^31^P NMR spectra can be assigned, in order of decreasing *δ*_iso_, as P(OGa)_4_, P(OGa)_3_(OAl), P(OGa)_2_(OAl)_2_, P(OGa)(OAl)_3_ and P(OAl)_4_ and, as shown in Fig. 9b, their integrated intensities match almost perfectly the intensities that would be expected for a random distribution of Al and Ga over the metal sites. This is a surprising observation, given that in the as-made materials the cations were not randomly distributed between the octahedral and tetrahedral sites. However, given that the single P site in the calcined AlGaPOs originated as P1, P2 and P3 in the as-made materials, the observed signal must be a superposition of signals with the NNN cation distributions of all three starting P sites. As can be seen in Fig. 9c, comparing the experimental intensities with those predicted for a model with the cation distribution determined from experiment in the as-made AlGaPOs, it is difficult to distinguish visually between this non-random cation distribution and a wholly random cation distribution. The mean absolute error between experimental and predicted peak intensities is 1.6% for the random model and 3.2% for the non-random model, but the estimated error in the experimental values is on the same order of magnitude, at *ca.* 1%. The overall cation distribution cannot, therefore, be determined directly from the integrated intensities of the ^31^P NMR signals in the calcined materials, despite the clear evidence for the non-random cation distributions in the parent as-made AlGaPOs. This curious finding suggests that other “randomly distributed” calcined framework materials might also have a more heterogeneous cation distribution than first thought, which may impact on their physical or chemical properties, such as in catalysis where Al and Si distribution in zeolites is being increasing recognised as modifying reactivity.^35^ Another possibility is that there some breaking of T–O bonds that could then allow redistribution of the Al and Ga; this would be consistent with recent work by Ashbrook and co-workers, who have used ^17^O NMR spectroscopy to show that even at room temperature it is possible to exchange O atoms from H_2_O into Si–O–Si linkages (as well as Si–O–Al) in aluminosilicate zeolites.^36–39^ The mixing energies calculated for the calcined solid solution, shown in Fig. 10, are all within 0.07 eV (≈7 kJ mol^−1^ if the formula unit is still M_6_P_6_O_24_ as in the discussion for the as-made materials above), which confirms that there is no preferred ordering for Al or Ga. As such, given a kinetically allowed pathway, rearrangement of Al and Ga at the elevated temperature of calcination could lead to a more random distribution of cations than observed in the as-made materials. However, we note that the mechanism(s) exploited by Ashbrook and co-workers^36–39^ involve the presence of H_2_O, which would be chemically unlikely under our high-temperature calcination conditions.

## Experimental and computational details

Mixed-metal Al–Ga oxide precursors were synthesised using varying concentrations of Al(acac)_3_ and Ga(acac)_3_ (acac = acetylacetonate). The required molar equivalents of Al(acac)_3_ (Sigma-Aldrich) and Ga(acac)_3_ (Sigma-Aldrich) (12 mmol total) were added to 55 mL propan-2-ol and stirred at room temperature for 30 min before heating in a fan assisted oven at 240 °C for 24 h. After cooling, the mixture was centrifuged and washed with acetone before drying overnight at 70 °C. Samples of γ-(Al_*x*_Ga_1−*x*_)_2_O_3_ were prepared with *x* = 0.25, 0.5 and 0.75. Note that although these mixed-metal oxides were prepared using a modified version of the procedure of Cook *et al.*^14^ (who used aluminium isopropoxide as the Al precursor), ^27^Al magic-angle spinning (MAS) NMR spectra (shown in Fig. S1†) indicate a similar ratio of octahedral and tetrahedral sites occupied by Al in materials prepared using either Al(acac)_3_ or Al(^i^PrO)_3_.

To synthesise the AlGaPOs, 0.25 g of γ-(Al_*x*_Ga_1−*x*_)_2_O_3_ was weighed into a 20 mL Teflon^®^ autoclave liner. H_3_PO_4_ (85%, Fisher Scientific), deionised water, the SDA precursor, 1-methylimidazole (99%, Sigma-Aldrich) and, lastly, HF (40%, Fluka) were then added. The additions were made to achieve a molar ratio of 1 γ-(Al_*x*_Ga_1−*x*_)_2_O_3_ : 2H_3_PO_4_ : 1 HF : 60H_2_O : 1.7 SDA. A magnetic stirrer was used to stir the reaction mixture at room temperature for at least 1 h (note that at shorter gel aging times the GaPO end member forms a different material, GaPO-34A^40^ so aging times of >1 h were also used to prepare the AlGaPOs). The autoclave was then assembled, sealed and placed in a fan-assisted oven at 170 °C for 24 h.

AlPO-34(mim) was prepared using a similar method but with hydrated alumina (Al_2_O_3_·1.9H_2_O, Sasol) as the metal source. The reaction mixture was stirred for 1.5 h before being heated at 170 °C for 10 days to produce a highly crystalline sample for structure determination by PXRD.

High-resolution PXRD data for structure solution of AlPO-34(mim) were recorded on Beamline I11 of Diamond Light Source, UK.^41^ The finely ground powder was loaded into a 0.1 mm diameter capillary and data recorded with an X-ray wavelength of 0.826855 Å using a position sensitive detector to avoid sample damage. All steps of the structural determinations (extractions of the peak positions, pattern indexing, simulated annealing processes to localise the organic template, difference Fourier map calculations and Rietveld refinement) were carried out with the TOPAS 5.0 program.^42^

Simultaneous TGA and differential scanning calorimetry (DSC) were performed using a Mettler Toledo TGA/DSC 1 instrument. Approximately 10 mg of each powder was loaded into separate alumina crucibles and data recorded on heating in air to 800 °C at 10 °C min^−1^. Prior to the measurement the material was dried in air at 70 °C overnight.

Solid-state NMR spectra were recorded either in house on Bruker Avance III spectrometers equipped with a 9.4 or 14.1 T wide-bore superconducting magnet or at the UK High-Field Solid-State NMR Facility on a Bruker Avance NEO spectrometer equipped with a 20.0 T wide-bore superconducting magnet. Further experimental details are given in the Table S1.† Chemical shifts are reported in ppm relative to Si(CH_3_)_4_ (^13^C), CFCl_3_ (^19^F), 1.1 M Al(NO_3_)_3_ in D_2_O (^27^Al), 85% H_3_PO_4_ (^31^P) and 1.1 M Ga(NO_3_)_3_ in D_2_O (^71^Ga) using secondary solid references of l-alanine (**C**H_3_ = 20.5 ppm), PTFE (C**F**_2_ = −122.7 ppm), **Al**(acac)_3_ (*δ*_iso_ = 0.0 ppm), B**P**O_4_ (−29.6 ppm) and **Ga**PO_4_ berlinite (*δ*_iso_ = 111.1 ppm).

Geometry optimisations and calculation of NMR parameters were carried out using the CASTEP DFT code (version 19.11),^43^ employing the GIPAW algorithm,^44^ to reconstruct the all-electron wave function in the presence of a magnetic field. The initial structures were taken from the literature or XRD refinements in this work. Calculations used the GGA PBE functional, with core–valence interactions described by ultrasoft pseudopotentials,^45^ which were generated on the fly, accounting for scalar relativistic effects by using ZORA.^46^ A modified pseudopotential was used for Ga, as described by Cook *et al.*^14^ A planewave energy cut-off of 60 Ry was used, and integrals over the Brillouin zone were performed using a Monkhorst–Pack grid with a *k*-point spacing of 0.04 2π Å^−1^. Dispersive interactions were reintroduced using the scheme of Tkatchenko and Scheffler (TS)^47^ as implemented by McNellis *et al.*^48^ Calculations were carried out on the St Andrews High Performance Computing Resource. For each solid solution (containing different SDA orientations – see the ESI† for more details), a complete set of 36 symmetry unique structural models was generated using the SOD program,^29^ with each structure then optimised using the parameters above. For the calcined structures, the higher average symmetry led to a complete set of 14 symmetry unique structures. Some additional input structures (see above and the ESI†) were generated manually and optimised as described above.

The calculations generate the diagonalised magnetic shielding tensor in the principal axes system, **σ**. From this, the isotropic shielding is given by *σ*_iso_ = (1/3) Tr{**σ**}, and the isotropic chemical shift, *δ*_iso_, by −(*σ*_iso_ − *σ*_ref_), where *σ*_ref_ is a reference shielding. Further details on the referencing of calculations are provided in the ESI.† The magnitude of the quadrupolar coupling constant is given by *C*_Q_ = *eQV*_ZZ_/*h*, where *Q* is the nuclear quadrupole moment (for which a value of 146.6 mb was used for ^27^Al).^49^ The asymmetry parameter is given by *η*_Q_ = (*V*_XX_ − *V*_YY_)/*V*_ZZ_.

## Conclusions

We report for the first time the synthesis and detailed characterisation of AlGaPOs, with the case study of the (Al_*x*_Ga_1−*x*_PO_4_)_3_·F·mim solid solution, prepared using mixed-metal oxide precursors. These AlGaPO-34(mim) materials were characterised using an NMR crystallographic approach, with particular focus on the distribution of the framework Al and Ga cations. We also prepared the new AlPO-34(mim) material for comparison, which is structurally similar to other as-made forms of AlPO-34. Its NMR spectra are consistent with those of AlPO-34 prepared with different organic SDAs.^17^

Although the crystal structures of the end members were well characterised, constructing structural models for the (Al_*x*_Ga_1−*x*_PO_4_)_3_·F·mim solid solution is not trivial owing to the fact that the three distinct metal sites may have different Al/Ga composition, the SDA is in a different orientation in each end member, and, while TGA and X-ray diffraction both indicate that no water was present in the AlPO-34(mim), a partially-occupied water site was located by diffraction for GaPO-34(mim). A combination of NMR spectroscopy and DFT calculations revealed that the Al in the phosphate framework of AlGaPO-34(mim) has a strong preference for octahedral coordination, whereas Ga has a preference for tetrahedral coordination. Indeed, this preference (of ∼20 kJ mol^−1^ rather than ∼5 kJ mol^−1^ in the parent metal oxides) leads to a large deviation from a random distribution of Al and Ga within the as-made materials. This is most clearly evidenced in the ^31^P NMR spectra, where the lineshapes observed for P1 can be rationalised only by comparison with chemical shifts calculated by DFT for the lowest energy structures (*i.e.*, those which maximise the number of octahedral Al).

While the energetics of the framework cation distribution did not appear to be particularly influenced by the precise arrangement of extra framework species within the pores (*i.e.*, SDA orientation and any water), the SDA was shown to favour the formation of hydrogen bonds either to water (if present) or to framework O atoms, with the geometry of the hydrogen bond to the framework O depending on whether this is Al–O–P or Ga–O–P. However, the rather flat potential energy surface for the pore contents is likely to contribute to dynamic behaviour of the SDA and water.

Upon calcination of the AlGaPOs, although the ^31^P MAS NMR spectra are consistent with a random distribution of Al and Ga across the (now all tetrahedral) metal sites, there is no expectation that thermal treatment alone should redistribute the Al and Ga. It is shown, however, that the non-random distribution of cations proved to exist in the as-made AlGaPO-34(mim) materials would also lead to a pattern of peak intensities similar to that seen for a random distribution for the calcined materials. These findings highlight the importance of fully characterising the (often lower symmetry and more complicated) as-made materials in addition to the calcined materials, despite the latter being generally of greater relevance to practical applications.

The distribution of cations in aluminosilicate frameworks has already been shown to affect their reactivity and can be controlled or modified post synthesis in a variety of ways.^35,50–52^ As such, probing the precise distribution of Al and Ga in these calcined AlGaPO frameworks by, for example, ^17^O isotopic enrichment and NMR spectroscopic experiments and pair-distribution function (PDF) analysis of diffraction data, is likely to provide a fruitful avenue of future research.

## Data availability

The research data supporting this publication can be accessed in ref. 53.^53^

## Author contributions

The manuscript was written through contributions of all authors.

## Conflicts of interest

There are no conflicts to declare.

## Supplementary Material

SC-015-D3SC06924A-s001

SC-015-D3SC06924A-s002
