# Supplementary material for: Site-directed cation ordering in chabazite-type AlxGa1−xPO4-34 frameworks revealed by NMR crystallography
Source: Chem Sci. 2024 Feb 14;15(12):4374–85. doi: 10.1039/d3sc06924a (PMC10952087; doi:10.1039/d3sc06924a)
Supplement: SC-015-D3SC06924A-s001 [file SC-015-D3SC06924A-s001.pdf]

Electronic Supplementary Material (ESI) for Chemical Science.
This journal is © The Royal Society of Chemistry 2024

Site-Directed Cation Ordering in Chabazite-Type $Al_xGa_{1-x}PO_4$-34 Frameworks Revealed by NMR Crystallography

Daniel M. Dawson,[1] Jasmine A. Clayton,[2] Thomas H. D. Marshall,[2] Nathalie Guillou,[3] Richard I. Walton[2] and Sharon E. Ashbrook[1]

1. School of Chemistry, EaStCHEM and St Andrews Centre for Magnetic Resonance, University of St Andrews, North Haugh, St Andrews KY16 9ST, UK.
2. Department of Chemistry, University of Warwick, Coventry, CV4 7AL, UK.
3. Institut Lavoisier, UMR CNRS 8180, Université de Versailles St-Quentin-en-Yvelines, Université Paris-Saclay, 78035 Versailles, France.

Supporting Information

S1. Further Details of Solid-State NMR Experiments
S2. Additional Characterisation of γ-$(Al_xGa_{1-x})_2O_3$ Precursors
S3. Additional Characterisation of AlPO-34(mim)
S4. Thermogravimetric Analysis of AlGaPO-34(mim)
S5. Additional Solid-State NMR Spectroscopic Characterisation of AlGaPO-34(mim)
S6. Additional Discussion of the DFT Calculations
S7. Additional Analysis of the Calcined AlGaPO-34 Series
S8. References

S1. Further Details of Solid-State NMR Experiments

Table S1 presents further information on the acquisition parameters for the solid-state nuclear magnetic resonance (NMR) experiments carried out in this work.

Table S1. Solid-state NMR experimental details: external field strength (B_0), MAS rate (ν_R), number of transients for signal averaging (NS) and recycle interval (D1).

Nucleus	Sample(s)	Pulse seq.	B_0 / T	ν_r / kHz	NS	D1 / s
^{13}C	As-made	CP MAS[a]	9.4	12.5	1024	3
^{19}F	As-made[b]	spin echo[c]	14.1	40	16 to 800	60
	GaPO-34(mim)[S1]	MAS	14.1	25	16	3
^{27}Al	γ-$(Al,Ga)_2O_3$	MAS[d]	9.4	14	256 to 2048	1
	As-made	MAS[d]	9.4	14	128	1
	As-made[e]	3QMAS[f]	9.4	14	24	1
	AlPO-34(mim)	3QMAS[f]	9.4	14	480	0.5
	Calcined	MAS	20.0	50	128 to 512	0.5
^{31}P	As-made	MAS	9.4	14	16	60
	Calcined	MAS	20.0	50	8 to 128	30
^{71}Ga	As-made	MAS[g]	20.0	50	5120 to 122880	0.5
	Calcined	MAS[g]	20.0	50	10240 to 40960	0.5

a. ^{1}H-^{13}C cross-polarisation (CP) MAS experiments used a spin lock pulse (ramped for ^{1}H) of 1 ms. TPPM-15 decoupling of ^{1}H ($\nu_1 \approx 100$ kHz) was applied during acquisition.

b. All as-made samples except for GaPO-34(mim)

c. Rotor-synchronized with $\tau = 25$ μs.

d. A pulse with a short flip angle ($\beta \approx 9°$) was used to ensure quantitative results.

e. All as-made samples except for AlPO-34(mim).

f. Recorded using an amplitude-modulated z-filtered pulse sequence with 128 t_1 increments of 71.43 μs. Spectra were sheared and referenced in the indirect dimension according to the convention in Ref. S2.

g. A pulse with a short flip angle ($\beta \approx 23°$) was used to ensure quantitative results.

S2. Additional Characterisation of γ-$(Al_xGa_{1-x})_2O_3$ Precursors

The ^{27}Al MAS NMR spectra of the γ-$(Al_xGa_{1-x})_2O_3$ precursors used in this work are shown in Figure S1a. Spectral integration provides the amounts of Al^{IV} and Al^{VI}, and these values are consistent with values reported by Cook *et al.*,[S3] who used $Al(^iPrO)_3$ as the Al source rather than the $Al(acac)_3$ used here. The values reported by Cook *et al.* (originally presented in Figure 8a of Ref. S3) are shown in Figure S1b (red and blue lines) with the values obtained in the present work shown as green crosses.

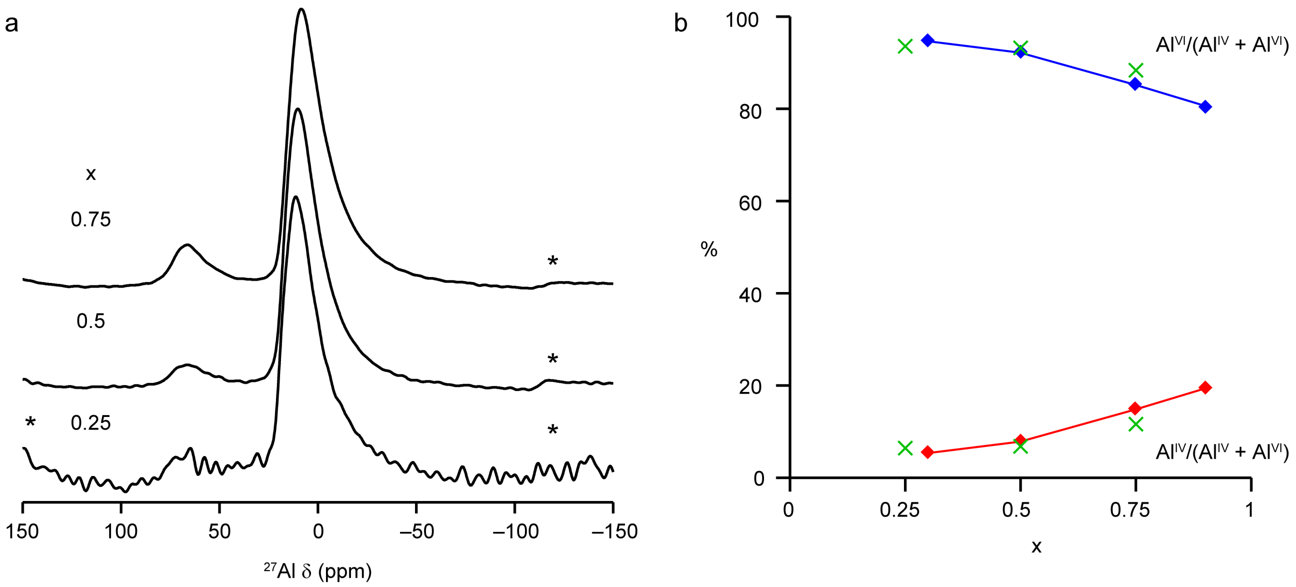

Figure S1. (a) ^{27}Al (9.4 T, 14 kHz MAS) NMR spectra of γ-$(Al_xGa_{1-x})_2O_3$ prepared using $Al(acac)_3$ as the Al source. (b) Plot of the proportion of Al^{IV} and Al^{VI} in the mixed-metal oxides (green crosses) compared with values reported by Cook *et al.*[S3] for the analogous oxides prepared with $Al(^iPrO)_3$ as the Al source (shown in red for Al^{IV} and blue for Al^{VI}).

S3. Additional Characterisation of AlPO-34(mim)

Figure S2 shows SEM images to confirm AlPO-34(mim) is polycrystalline.

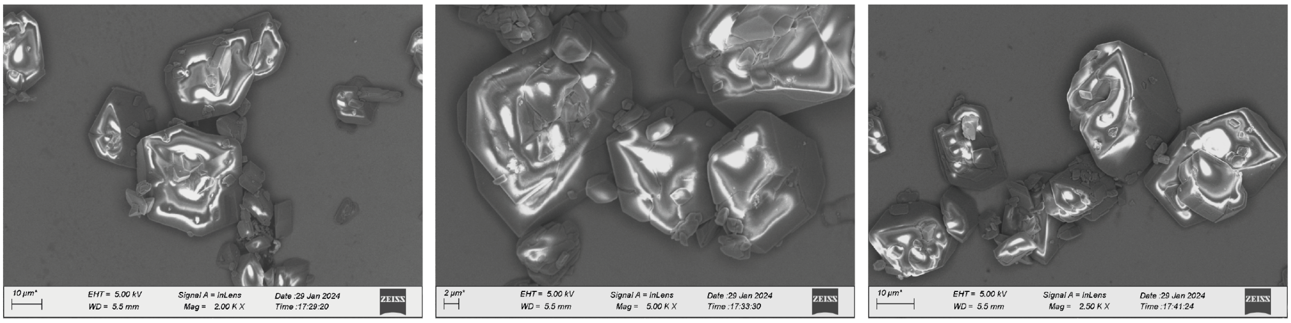

Figure S2. SEM images of AlPO-34(mim).

Figure S3 shows the two-dimensional ^{27}Al 3QMAS NMR spectrum of AlPO-34(mim) after shearing, along with the sum projection on δ_1 and slices extracted parallel to δ_2 for each of the three signals.

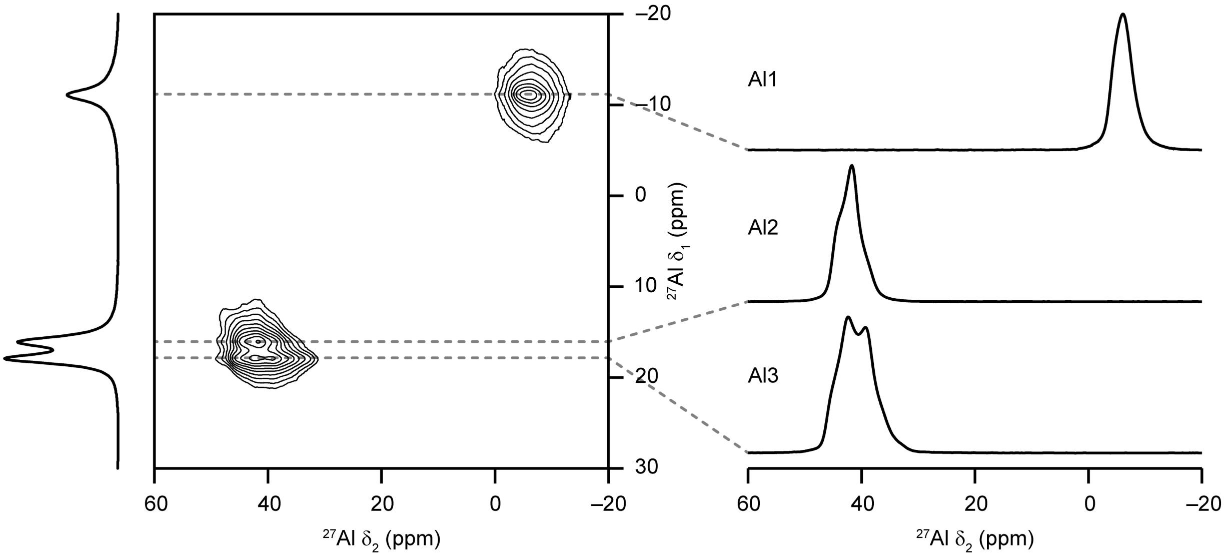

Figure S3. ^{27}Al (9.4 T, 14 kHz MAS) 3QMAS NMR spectrum of AlPO-34(mim) showing the sum projection onto δ_1 and slices extracted parallel to δ_2 for the three signals.

S4. Additional Characterisation of AlGaPO-34(mim)

Figure S4 shows the powder X-ray diffraction (PXRD) patterns for the five samples studied, confirming pure phase chabazite-type materials are present in each case.

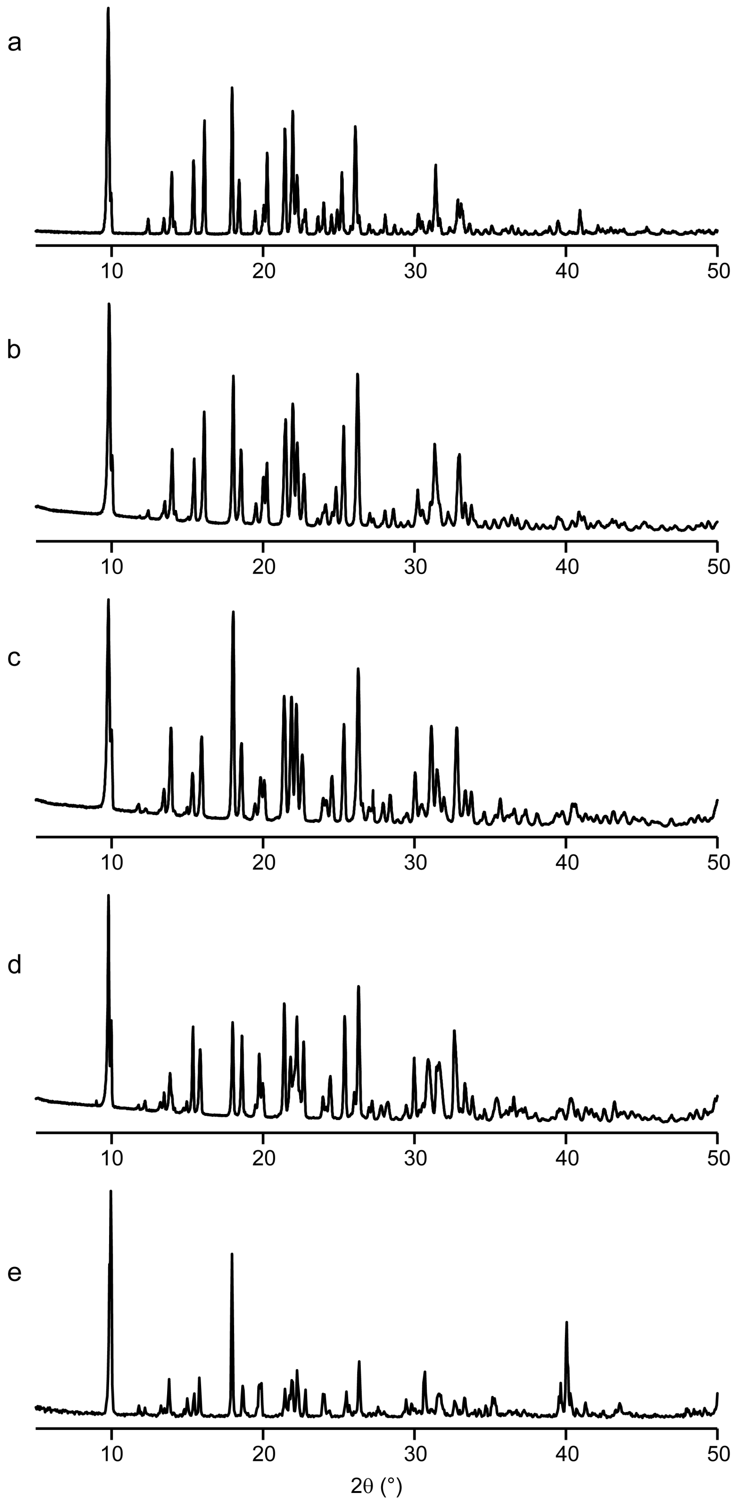

Figure S4. PXRD patterns for the five $Al_xGa_{1-x}PO$-34(mim) samples studied, with (a) x = 1, (b) x = 0.75, (c) x = 0.5, (d) x = 0.25 and (e) x = 0.

Figure S5 shows the thermogravimetric analysis (TGA) and differential scanning calorimetry (DSC) traces for AlPO-34(mim) and the AlGaPO-34(mim) samples. We previously reported the equivalent data for GaPO-34(mim)[S1,S4] and data recorded for this work, shown in Figure S5e is consistent with the earlier work. There is a slight difference in the overall mass loss for the two GaPO-34(mim) samples, which is ascribed to different amounts of absorbed water (0.65 per formula unit in Ref. S1 and 0.15 per formula unit here). It is interesting to note that in GaPO-34(mim), the calcination has previously been shown to occur in two stages,[S4] with loss of HF occurring at slightly lower temperature than loss of the 1-methylimidazole. Such a two-stage process could be proposed to occur for the most Ga-rich AlGaPO (see Figure S5d), but it was beyond the scope of the present work to investigate the calcination mechanism for AlGaPOs in further detail.

Table S2 shows the final experimental formulas for the five samples studied, determined using a combination of ICP-MS and TGA data.

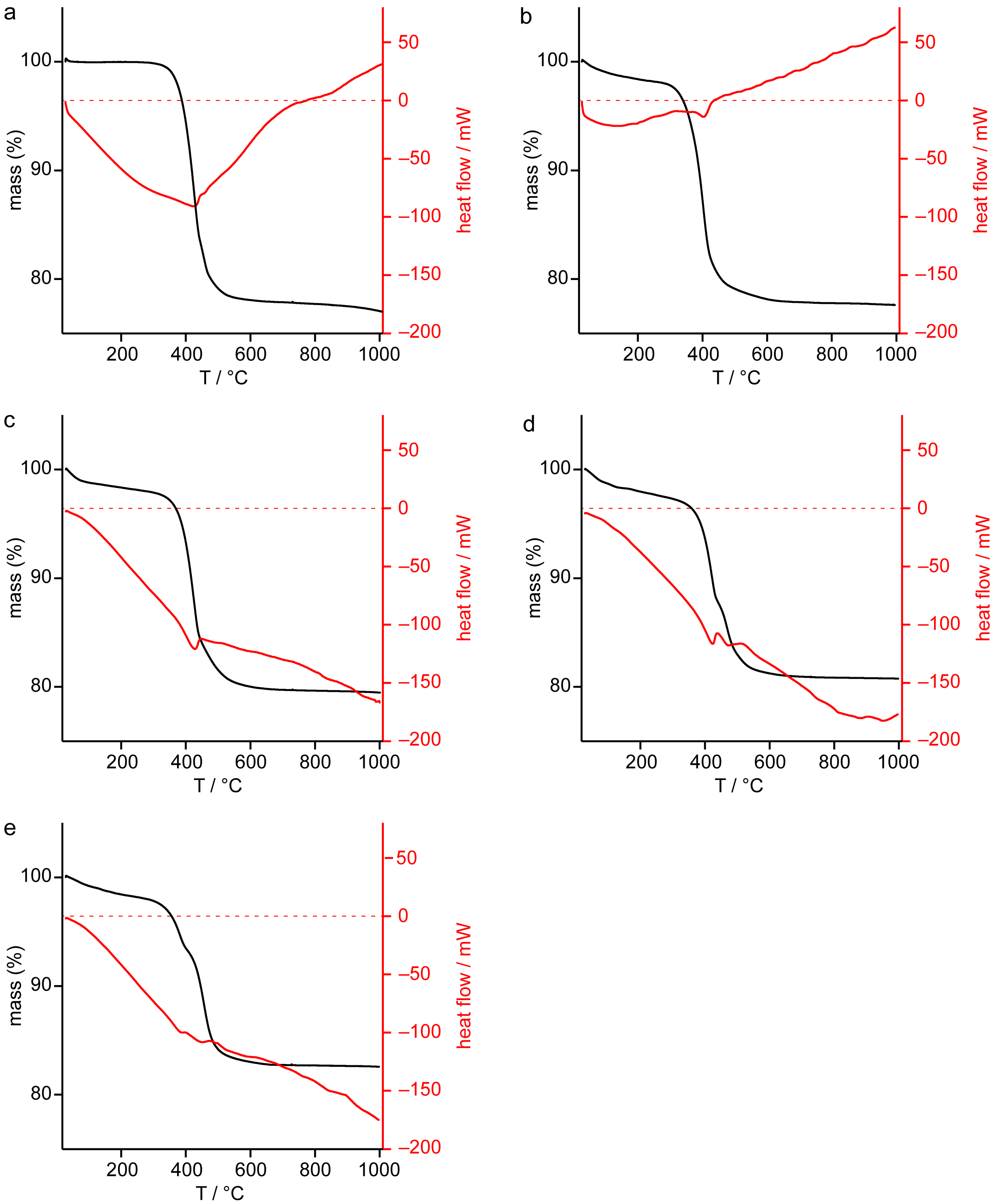

Figure S5. Thermogravimetric analysis (black) and differential scanning calorimetry (red) traces for the five $Al_xGa_{1-x}PO$-34(mim) samples studied, with (a) x = 1, (b) x = 0.75, (c) x = 0.5, (d) x = 0.25 and (e) x = 0. The dashed red line indicates a heat flow of zero.

Table S2. Composition of the $(Al_xGa_{1-x})_3P_3O_{12}\cdot F\cdot mim\cdot yH_2O$ materials determined from ICP-MS and TGA data.

x	Formula	Total mass loss (%)	
		Calc.	Obs.[a]
1	$Al_3P_3O_{12}\cdot F\cdot mim$	21.8	22.3
0.75	$(Al_{0.73}Ga_{0.27})_3P_3O_{12}\cdot F\cdot mim\cdot 0.68H_2O$	21.6	22.2
0.5	$(Al_{0.45}Ga_{0.55})_3P_3O_{12}\cdot F\cdot mim\cdot 0.60H_2O$	21.0	20.5
0.25	$(Al_{0.21}Ga_{0.79})_3P_3O_{12}\cdot F\cdot mim\cdot 0.51H_2O$	20.5	19.2
0	$Ga_3P_3O_{12}\cdot F\cdot mim\cdot 0.15H_2O$	18.7	17.5

a. Mass lost on combustion.

S5. Additional NMR Spectroscopic Characterisation of AlGaPO-34(mim)

Figure S6a shows the δ_1 projections of the Al^{IV} signals from the 3QMAS NMR spectra of the Al-containing AlGaPO samples. While the signal-to-noise ratio is clearly poorer for the sample containing least Al, spectral integration is still possible, assuming that the lineshapes in the isotropic dimension can be described by Gaussian-Lorentzian lines. Figure S6b shows a plot of the ratio of the integrated intensities of the Al2 and Al3 signals as a function of composition, which suggests that neither site is particularly favoured for Ga substitution. Note that the MQMAS experiment is not quantitative, with excitation efficiency varying with C_Q, such that even in the AlPO-34(mim) end member the ratio of signal from Al3/Al2 is not exactly 1.

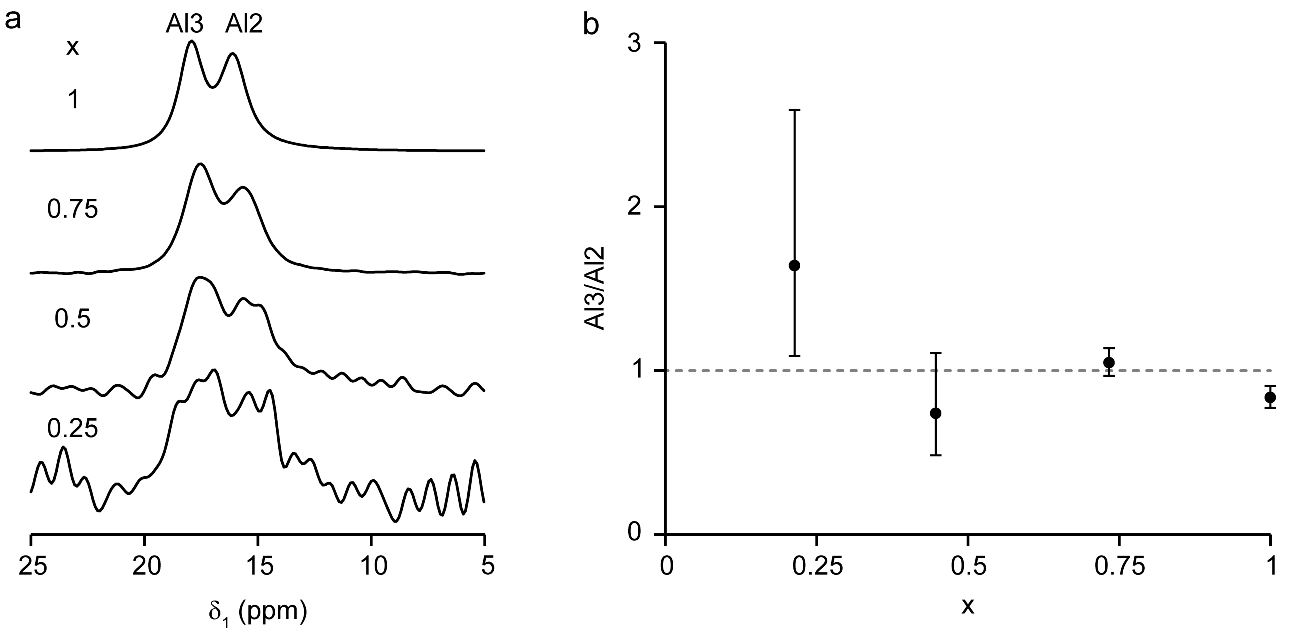

Figure S6. (a) δ_1 projections of ^{27}Al (9.4 T, 14 kHz 3QMAS) NMR spectra of as-made $Al_xGa_{1-x}PO_4$-34(mim) with x = 0.25, 0.5, 0.75 and 1.0, showing only the signals for Al^{IV}. (b) Plot of the integrated intensity ratio of Al3/Al2 against composition, x, with error bars showing indicative errors of ±2% for x = 1 and 0.75, and ±10% for x = 0.5 and 0.25. The dashed line indicates Al3/Al2 = 1 and is shown as a guide to the eye.

Figure S7 shows expansions of the aromatic region of the ^{13}C CP MAS NMR spectra for the samples studied. While small changes are observed in peak positions, as shown in Figure S7b, these are not particularly systematic with composition and likely reflect a combination of SDA orientation effects and the varying degrees of hydration of the samples.

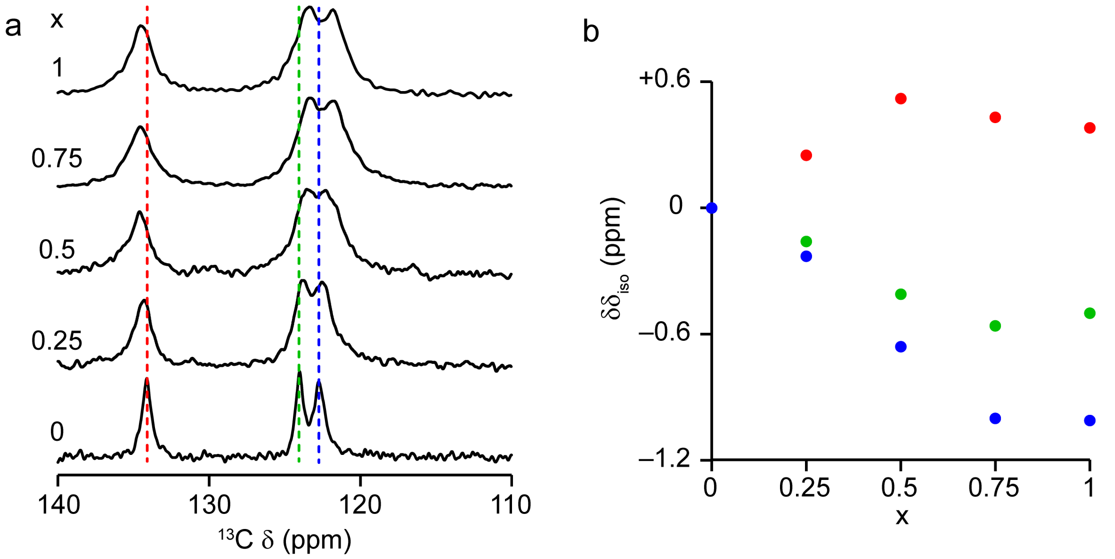

Figure S7. (a) Expansions of the aromatic region of the ^{13}C (9.4 T, 12.5 kHz CP MAS) NMR spectra of the $Al_xGa_{1-x}PO_4$-34(mim) samples. (b) Plot of the change in δ_{iso}, $\delta\delta_{iso}$, relative to GaPO-34(mim) against composition, x, for the three aromatic ^{13}C signals.

Figure S8a shows the integrated intensities of the three ^{31}P signals assigned to P1 in the $Al_xGa_{1-x}PO_4$-34(mim) samples, as a function of x. Using the binomial theorem, the relative intensities of the signals for a P with n P-O-Al linkages, p(nAl) can be expressed as:

$$p(nAl) = x^n(1-x)^{4-n} \ , \qquad \text{Equation S1}$$

where $0 \leq n \leq 4$. This model assumes a random distribution of Al and Ga on each of the four next-nearest neighbour sites, and it can be seen from Figure S8a that there is poor agreement with any of the experimental intensities. This is to be expected, however, since the ^{27}Al, ^{71}Ga and ^{19}F NMR spectra showed that the Al and Ga exhibit a preference for the octahedral and tetrahedral sites, respectively.

A more realistic model, assuming a random distribution of Al and Ga in the octahedral sites, with the composition of this site given as x_O, and a random distribution of Al and Ga in the tetrahedral sites with composition x_T, and bearing in mind that P1 has two P-O-M^{IV} and two P-O-M^{VI} (M = Al or Ga) linkages, leads to the following expressions.

For the distribution of Al on the two octahedral sites:

$$p(0Al^{VI}) = (1-x_O)^2 \ ,$$
$$p(1Al^{VI}) = 2x_O(1-x_O) \ ,$$

$p(2Al^{VI}) = x_O^2$. Equation S2

For the distribution of Al on the two tetrahedral sites:

$p(0Al^{IV}) = (1–x_T)^2$,

$p(1Al^{IV}) = 2x_T(1–x_T)$,

$p(2Al^{IV}) = x_T^2$. Equation S3

Therefore, for P1 overall,

$p(0Al) = (1–x_O)^2(1–x_T)^2$,

$p(1Al) = 2x_O(1–x_O)(1–x_T)^2 + 2x_T(1–x_O)^2(1–x_T)$,

$p(2Al) = 4x_O(1–x_O)x_T(1–x_T)^2 + x_O^2(1–x_T)^2 + x_T^2(1–x_O)^2$,

$p(3Al) = 2x_O^2x_T(1–x_T) + 2x_T^2x_O(1–x_O)$,

$p(4Al) = x_O^2x_T^2$, Equation S4

which, in the limit of $x_O = x_T$, is equivalent to Equation S1.

The values of x_O and x_T can be determined experimentally from the ^{19}F NMR spectra (for x_O) and a combination of x_O and the composition of the material from elemental analysis (for x_T). Figure S8b shows that Equation S4 yields predicted values for P1(3Al) and P1(4Al) in good agreement with the observed integrated intensities of the two signals at lower shift (compare the green and blue points and lines in Figure S8b). This observation suggests the assignment of the three observed signals as P1(0-2Al), P1(3Al) and P1(4Al), as discussed in the main text. Figures S8c and S8d show predicted integrated intensities for P1(0-2Al), P1(3Al) and P1(4Al) using Equation S1 and Equation S4, respectively.

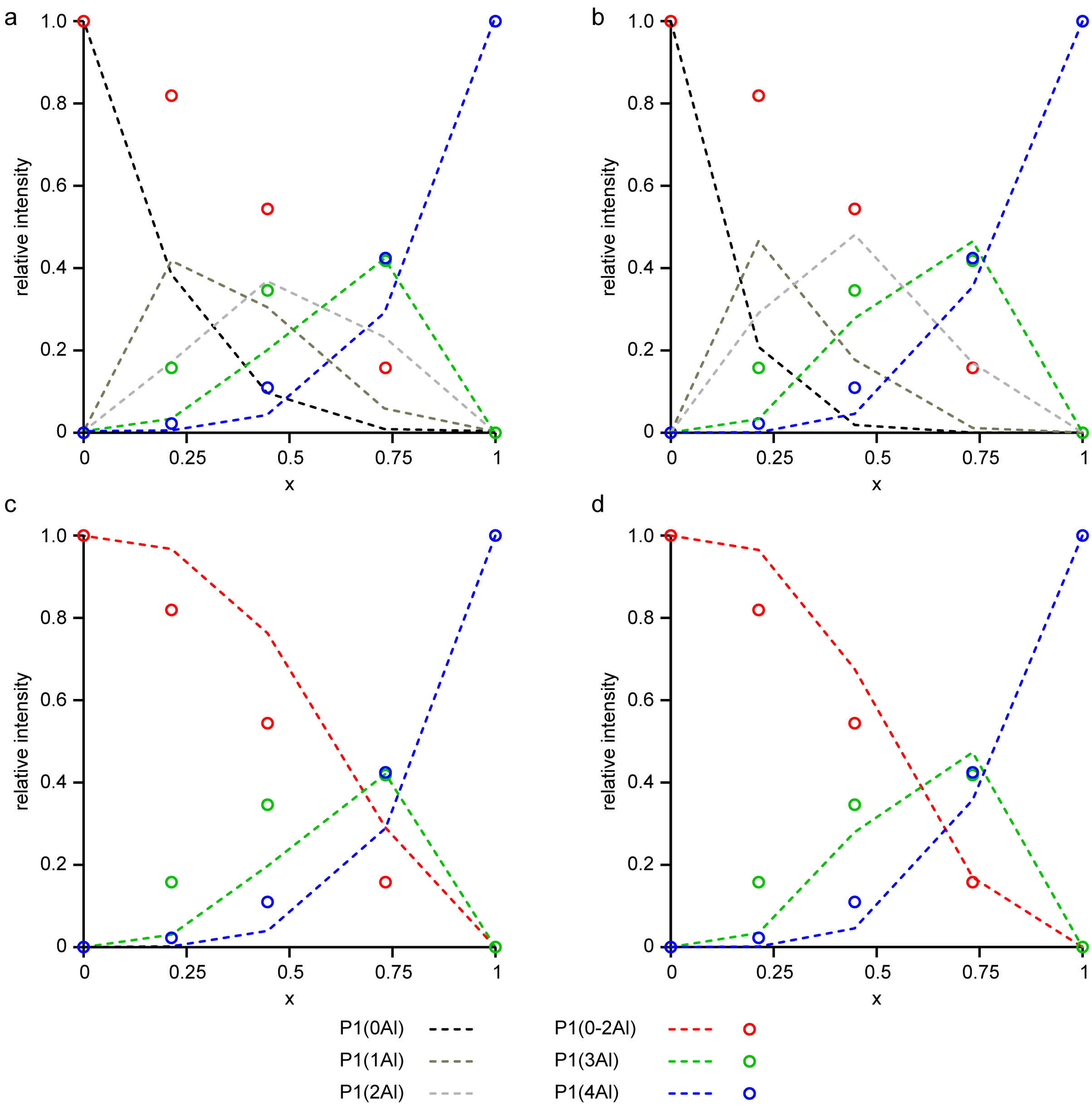

Figure S8. Plots of relative spectral intensity for P1 with n Al next-nearest neighbours against composition, x. In each case the open circles represent the experimental data and dashed lines indicate the intensities calculated (a) from Equation S1, (b), from Equation S4, (c) from Equation S1 assuming the lines for $0 \leq n \leq 2$ overlap and (d) from Equation S4, assuming the lines from $0 \leq n \leq 2$ overlap.

S6. Additional Discussion of the DFT Calculations

DFT calculations using CASTEP generate the diagonalised absolute magnetic shielding tensor in the principal axes system, **σ**, from which the isotropic magnetic shielding is given by

$$\sigma_{iso} = \frac{1}{3}\mathrm{Tr}\{\boldsymbol{\sigma}\} \quad . \qquad \text{Equation S5}$$

To compare calculated shielding with experimental chemical shifts, a reference value, σ_{ref}, is used, with

$$\delta_{iso} = -(\sigma_{iso} - \sigma_{ref}) \quad . \qquad \text{Equation S6}$$

A plot of calculated σ_{iso} against experimental δ_{iso} should yield a straight line with gradient of –1 and an intercept of σ_{ref}. Figure S9 shows such plots for ^{27}Al and ^{31}P, using experimental values from calcined AlPO-14, calcined AlPO-34, calcined GaPO-34 and $GaPO_4$ berlinite (noting that the latter two contain P but not Al). Note that, since the gradients of both lines differ significantly from –1, Equation S6 was modified to give

$$\delta_{iso} = \frac{(\sigma_{iso} - \sigma_{ref})}{m} \quad , \qquad \text{Equation S7}$$

where m is the gradient of the line. It is also worth noting that the points for ^{27}Al in AlPO-34(mim), particularly the octahedral Al1, lie on a line significantly different from the reference set. We interpret this discrepancy as possible evidence of either SDA dynamics in the as-made AlPO-34(mim), or that the sample used in the NMR experiments had absorbed some atmospheric water, which was not present in the crystal structure determination (and, hence, the DFT calculation). Table S3 shows the values of σ_{ref} and m used here to calculate the ^{27}Al and ^{31}P δ_{iso}.

For ^{19}F, calculated shifts were referenced using the experimental shifts of the two end members (–98.0 and –125.0 ppm for the GaPO-34(mim) and AlPO-34(mim), respectively) and the calculated shifts for the two anhydrous materials with the SDAs in the orientations shown in Figure S10. We note that the value of m obtained using this method deviates significantly from –1, as has previously been observed for ^{19}F in the literature.[S5]

Table S3. Values of reference shielding and gradient for ^{19}F, ^{27}Al and ^{31}P used in this work.

Nucleus	σ_{ref} (ppm)	m
^{19}F	7.4	–2.21
^{27}Al	567.84	–1.14
^{31}P	288.18	–1.13

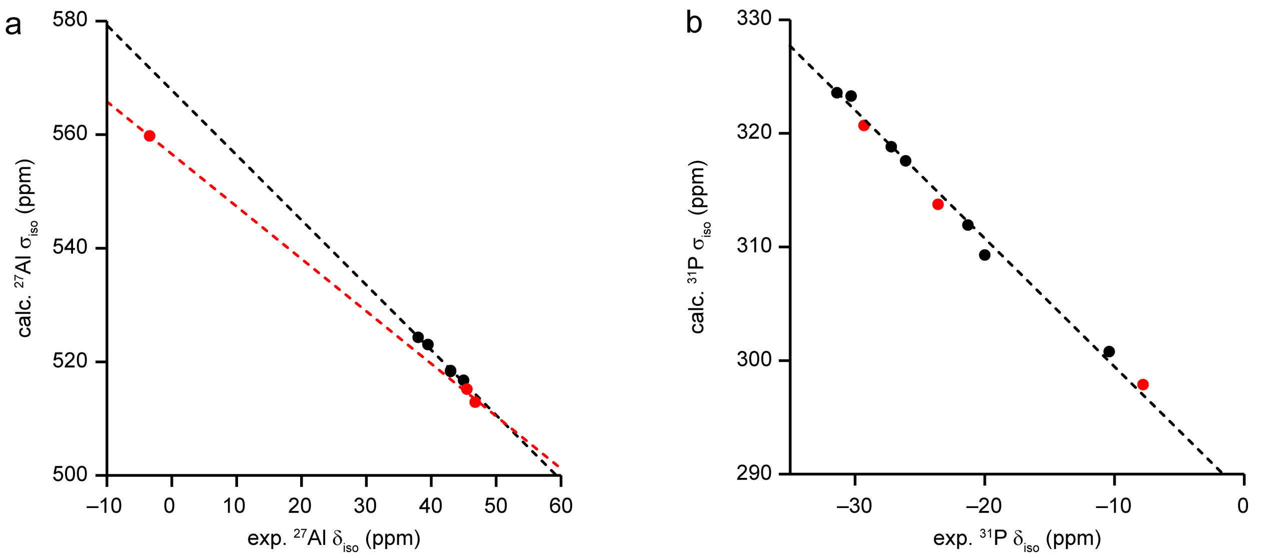

Figure S9. Plots of calculated σ_{iso} against experimental δ_{iso} used to reference calculated δ_{iso} for (a) ^{27}Al and (b) ^{31}P. Black points are for the reference data and red for AlPO-34(mim). Black dashed lines indicate the line of best fit for the reference data and in (a) the line of best fit for the AlPO-34(mim) data is shown in red.

The SOD program[S6] was used to generate two series of AlGaPO-34(mim) models with the SDA orientation either matching that of AlPO-34(mim) or GaPO-34(mim).[S7] In both cases, an anhydrous structure was considered (*i.e.*, the experimental structure for the AlPO and with the water molecule deleted for the GaPO). Figure S10 shows the parent structures for each series. The unit cells each contain six M sites (M = Al or Ga), leading to a total of 36 symmetry-distinct arrangements for substituting up to six Al into the GaPO framework (or *vice versa*) for each series. The main text describes results for series where Al was substituted into the dehydrated GaPO structure (Figure S10b) and Figures S11 and S12 compare the calculated mixing energies (as in Figure 6 of the main text) and calculated ^{31}P δ_{iso} (Figure 7a of the main text) for the two different series.

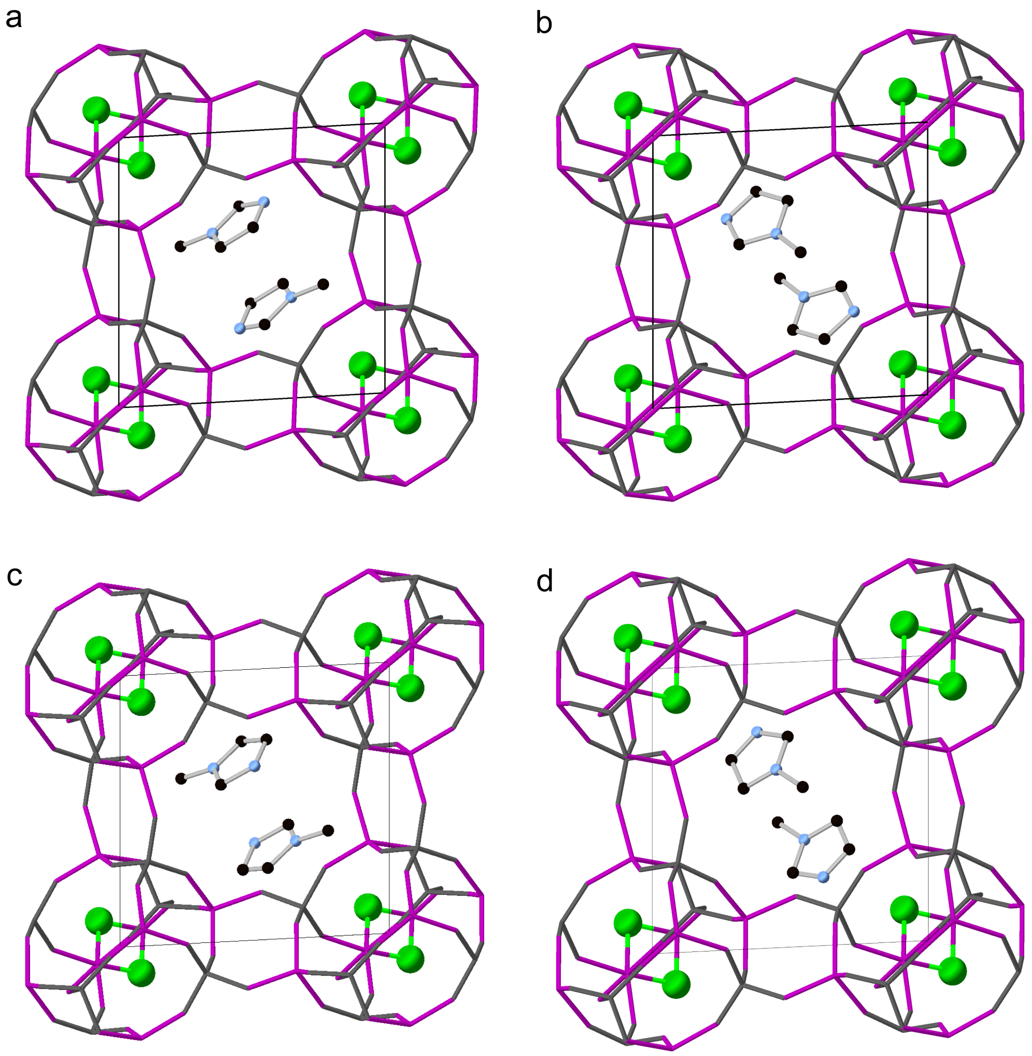

Figure S10. Parent framework structures for the series of models for AlGaPO-34(mim) with the SDA in the orientation of (a) the AlPO-34(mim) and (b) the GaPO-34(mim) end members and the (c) AlPO-34(mim) and (d) GaPO-34(mim) "reverse N" models (corresponding to a pseudo-C_2 rotation of the SDA about the H_3C–N bond). Structures are viewed down the crystallographic *c* axis, with C = black, N = blue, F = green, M = purple framework, P = grey framework, O and H atoms are hidden. Note the purple framework cation sites may be occupied by either Al or Ga, depending on the exact structural model considered.

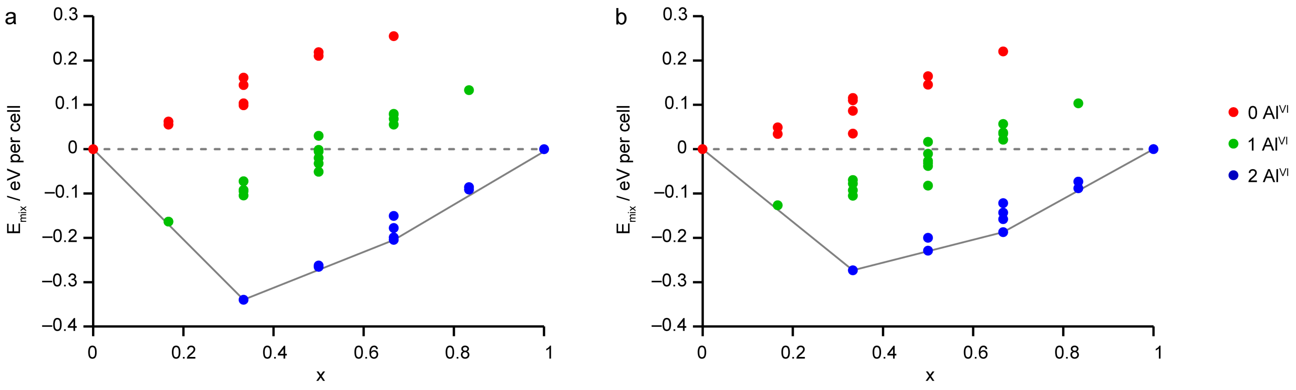

Figure S11. Plots of E_{mix} against x for the 36 distinct arrangements of Al and Ga in the AlGaPO-34(mim) structural models with the SDA in the orientation matching (a) the AlPO end member and (b) the anhydrous GaPO end member. The dashed grey line indicates E_{mix} = 0 and the solid grey line is the convex hull. Note that unlike Figure 6 of the main text, in this figure E_{mix} = 0 has been calculated separately for each series.

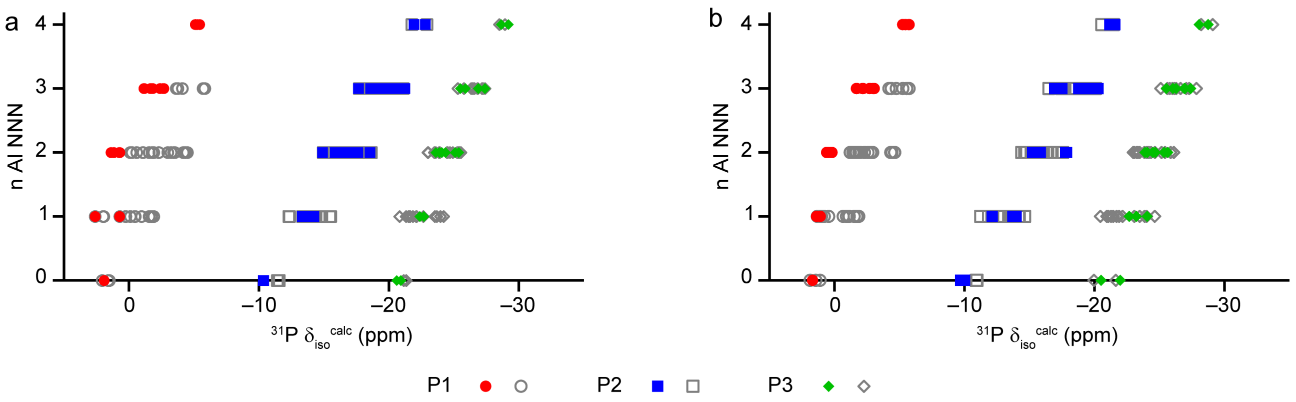

Figure S12: Calculated ^{31}P δ_{iso} for P sites with 0-4 Al NNN in the 36 distinct structural models for as-made AlGaPO-34 with the SDA in the orientation matching (a) the AlPO end member and (b) the anhydrous GaPO end member. Points corresponding to structures on or near the convex hulls (see Figure S11) are shown in colour, whereas the much less energetically favourable structures are shown in grey.

As a model for disorder of the SDA, structures with a pseudo-C_2 rotation of the molecule about the H_3C–N bond (effectively swapping the N on the 3 position for the C on the 4 position of the imidazole ring) were also considered. These "reverse N" structures again yield similar results with regards to the energetics of framework cation distributions, as can be seen in Figure S13. The full series of 36 structures each were not considered here as the results for substituting one or five Al into the parent GaPO structure (or *vice versa*) showed such similarity with the analogous results in Figure S11.

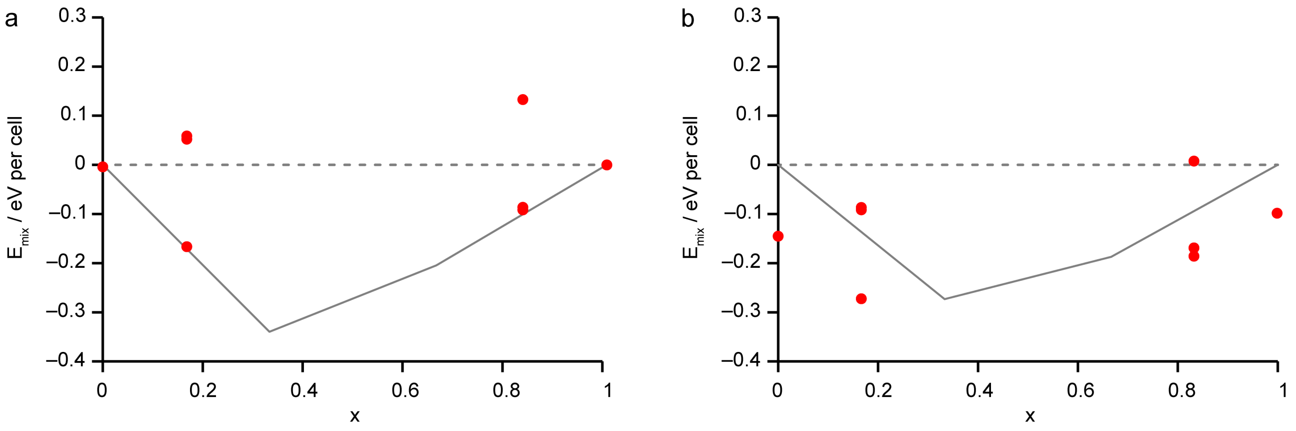

Figure S13. Plots of E_{mix} against x for 8 "reverse N" structures based on (a) the AlPO-34(mim) structure and (b) the dehydrated GaPO-34(mim) structure. The dashed grey line indicates $E_{mix} = 0$ for the crystallographically-determined C and N positions (for comparison with Figure S11) and the solid grey lines are the convex hulls shown in Figure S11. $E_{mix} = 0$ has been calculated separately in parts (a) and (b).

From Figure S13b it could be concluded that the experimental structure of the GaPO has the SDA in the incorrect orientation, as the reverse N models are lower in energy than those with the SDA in the experimentally determined orientation. However, the experimentally determined structure also contains a partially occupied molecule of water and, when this is occupied, as in Figure S14a, the reverse N models are less stable than those with the experimental orientation of the SDA, as shown in Figure S14b. As shown in Figure 8 of the main text, in the orientation indicated in the experimental structure solution the SDA is able to form an N–H···O hydrogen bond with the water, which is not possible in the reverse N structures. Therefore, it can be concluded that the orientation of any given SDA cation is likely to be heavily influenced by the presence of water in the same pore. However, notably, the most favoured metal sites for Al and Ga substitution do not change appreciably when the pore contents or SDA orientation are changed.

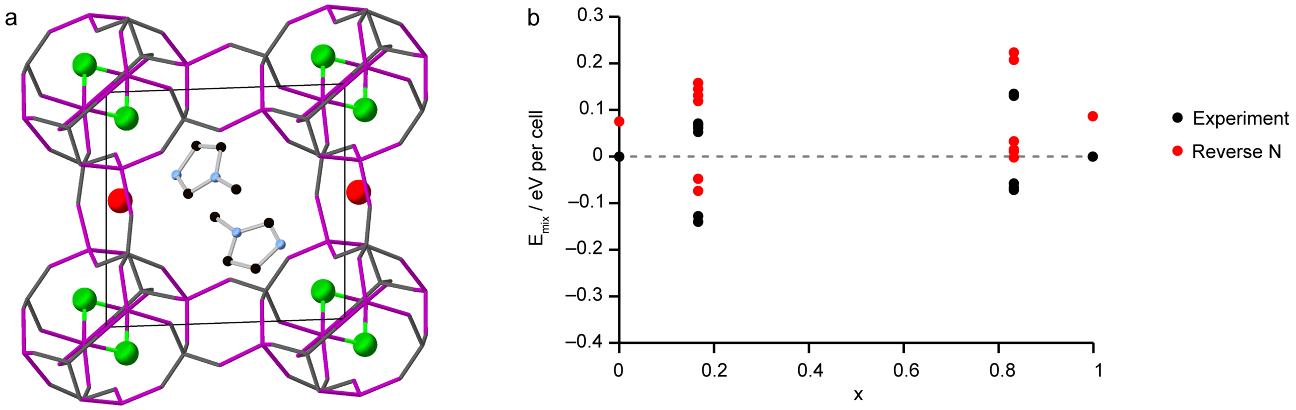

Figure S14. (a) Parent structure for the series of models for AlGaPO-34(mim) based on the GaPO-34(mim) structure with water present. The structure is viewed down the crystallographic *c* axis, with C = black, N = blue, F = green, M = purple wireframe, P = grey wireframe, O and H atoms are hidden, apart from the O atom of water, which is shown in red. (b) Plots of E_{mix} against x for 14 structures based on the experimental hydrated GaPO-34(mim) structure (closed circles) and the corresponding 14 "reverse N" models. The dashed grey line indicates E_{mix} = 0 for the crystallographically determined C and N positions.

Figure S15a shows the ^{19}F MAS NMR spectra of the AlGaPOs (reproduced from Figure 3a of the main text), overlaid with the calculated ^{19}F δ_{iso} for the 72 anhydrous structural models of the as-made mixed-metal materials generated by SOD as discussed above. The assignment of the signals as Al–F–Al, Al–F–Ga and Ga–F–Ga is unambiguous. Figures S15b and S15c show plots of the calculated δ_{iso} against the mean F–M bond length (M = Al, Ga) and M–F–M bond angle, respectively. It can be seen that, as proposed in the main text, the Al–F bond lengths are shorter and the Al–F–Al bond angles are slightly larger than the Ga–F and Ga–F–Ga bond lengths and angles, indicating that when Al is on the octahedral site, the M–F interaction is stronger and slightly more covalent than when Ga is on the octahedral site.

Figure S16 shows the calculated Mulliken charges for framework O atoms in the 36 anhydrous structural models of as-made AlGaPO-34 with the SDA in the orientation of the AlPO end member. There is clear separation between Al-O-P and Ga-O-P linkages, with Al-O-P oxygens being more negative than Ga-O-P oxygens (as would be expected for the slightly more ionic $AlPO_4$ framework). Additionally, for the Al-O-P linkages there is clear separation between Al^{IV}-O-P and Al^{VI}-O-P linkages, with the latter having a lower charge on the O atoms. Such separation is not observed for Ga^{IV}-O-P *vs* Ga^{VI}-O-P linkages, presumably as a consequence of the more covalent nature of Ga-O-P linkages in general. Table S4 summarises the numerical values of the Mulliken charges presented graphically in Figure S16.

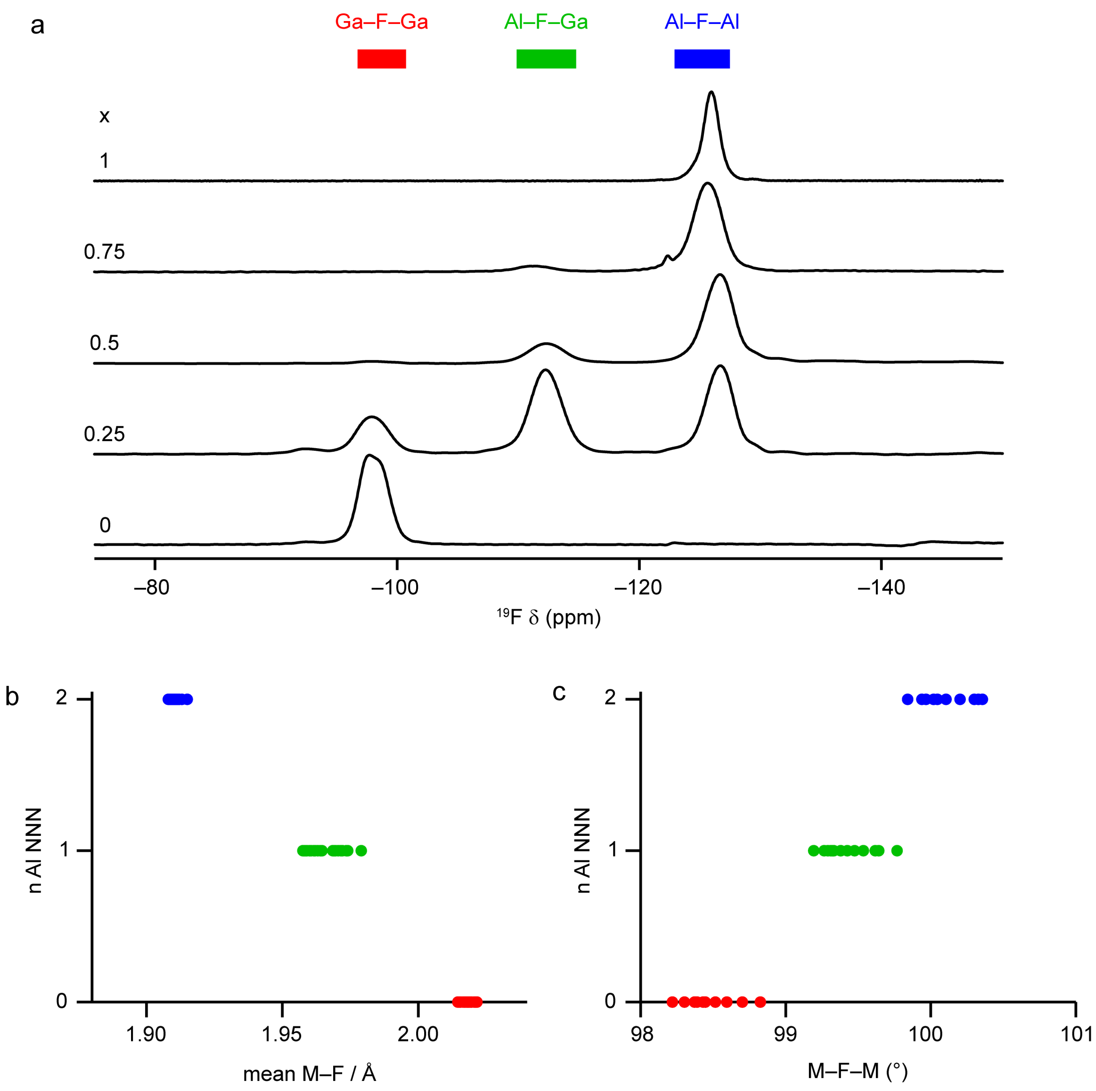

Figure S15. (a) ^{19}F (14.1 T, 25-40 kHz MAS) NMR spectra of the AlGaPO-34(mim) series, from Figure 3 of the main text, with the calculated ranges of ^{19}F δ_{iso} (from the two series of SOD-generated structures) indicated. (b and c) Plots of calculated ^{19}F δ_{iso} against (b) mean M–F bond length and (c) the M–F–M bond angle for the 72 optimised structural models of anhydrous AlGaPO-34(mim) generated by SOD.

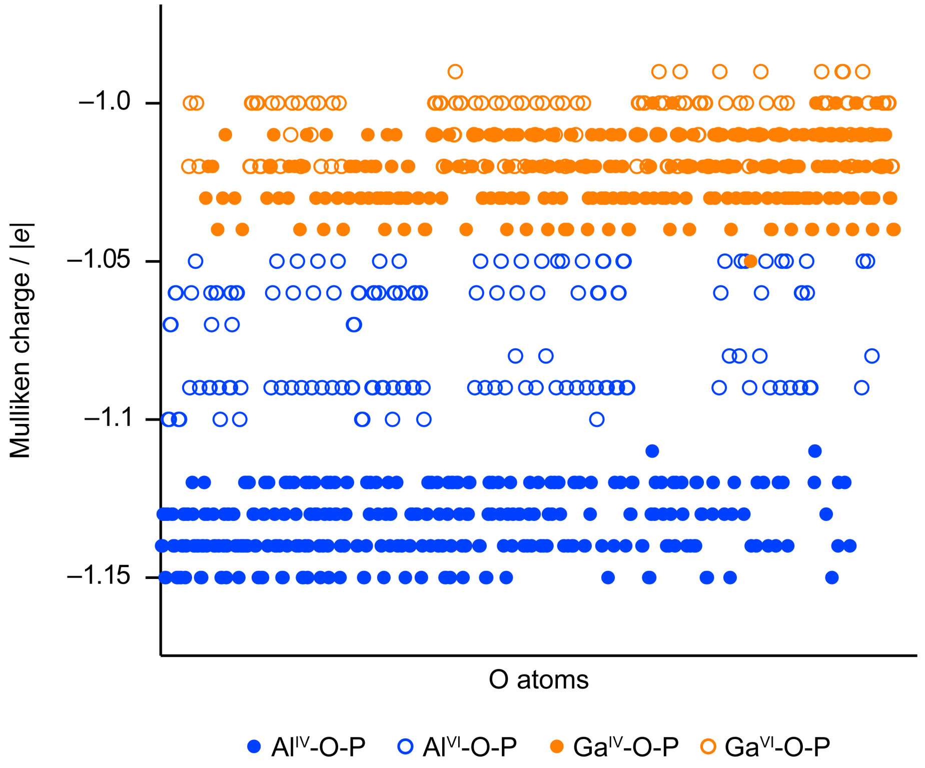

Figure S16. Plot of the calculated Mulliken charges on framework O atoms in the 36 anhydrous structural models of as-made AlGaPO-34 with the SDA in the orientation of the AlPO end member. The x axis is quantitatively meaningless but qualitatively corresponds to structures with a higher Al content to the left and a higher Ga content to the right.

Table S4. Calculated Mulliken charges for O atoms in Al-O-P and Ga-O-P linkages.

Linkage	Mulliken charge / |*e*|		
	minimum	mean (s.d.)[a]	maximum
Al-O-P	–1.15	–1.114 (0.031)	–1.05
Ga-O-P	–1.05	–1.017 (0.012)	–0.99
Al^{IV}-O-P	–1.15	–1.134 (0.010)	–1.11
Al^{VI}-O-P	–1.10	–1.074 (0.018)	–1.05
Ga^{IV}-O-P	–1.05	–1.022 (0.011)	–1.00
Ga^{VI}-O-P	–1.02	–1.007 (0.010)	–0.99

a. s.d. = standard deviation.

S7. Additional Analysis of the Calcined AlGaPO-34 Series

Figure S17 shows the ^{27}Al and ^{71}Ga MAS NMR spectra of the mixed-metal samples of calcined, dehydrated AlGaPO-34 as well as the ^{71}Ga NMR spectrum of the Ga end member. The ^{27}Al NMR spectrum of the Al end member was not recorded

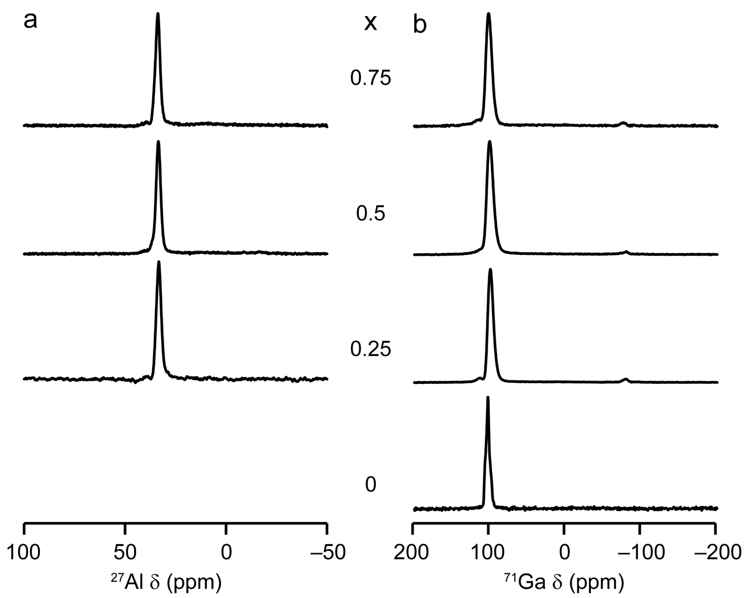

Figure S17. (a) ^{27}Al (20.0 T, 50 kHz MAS) and (b) ^{71}Ga (20.0 T, 50 kHz MAS) NMR spectra of the calcined, dehydrated AlGaPO-34 series.

As discussed in the main text, the ^{31}P MAS NMR spectra appear to show a nearly random distribution of Al and Ga on the metal sites, despite the as-made AlGaPOs showing significant deviation from random metal site occupancy. The relative intensities of the signals for a P with n P-O-Al linkages, p(nAl) is given by Equation S1 for a fully random cation distribution.

For a non-random cation distribution, a model based on the discussion above for the as-made materials can be constructed. Using the notation above for x_O as the composition of the octahedral sites and x_T as the composition of the tetrahedral sites leads to Equation S4 (above) for site P1 and the following expressions for sites P2 and P3 (both of which have one octahedral and three tetrahedral NNN cations).

For the one octahedral site:

$$p(0Al^{VI}) = (1 - x_O) \ ,$$
$$p(1Al^{VI}) = x_O \ . \qquad \text{Equation S8}$$

For the three tetrahedral sites:

$$p(0Al^{IV}) = (1-x_T)^3 \ ,$$
$$p(1Al^{IV}) = 3x_T(1-x_T)^2 \ ,$$
$$p(2Al^{IV}) = 3x_T^2(1-x_T) \ ,$$
$$p(3Al^{IV}) = x_T^3 \ . \qquad \text{Equation S9}$$

Therefore, for P2 and P3 overall,

$$p(0Al) = (1-x_O)(1-x_T)^3 \ ,$$
$$p(1Al) = x_O(1-x_T)^3 + 3x_T(1-x_O)(1-x_T)^2 \ ,$$
$$p(2Al) = 3x_Ox_T(1-x_T)^2 + 3x_T^2(1-x_O)(1-x_T) \ ,$$
$$p(3Al) = 3x_Ox_T^2(1-x_T) + x_T^3(1-x_O) \ ,$$
$$p(4Al) = x_Ox_T^3 \ , \qquad \text{Equation S10}$$

which, in the limit of $x_O = x_T$, is equivalent to Equation S1.

Bearing in mind that, in the calcined material, there is only one T site in the CHA framework, the observed ^{31}P NMR spectrum is a superposition of signals from P sites that were formerly P1, P2 and P3 in the as-made material. As such, for the same cation distribution as observed in the as-made material, the ^{31}P spectral intensities in the calcined material would be given by:

$$p(0Al) = [(1-x_O)^2(1-x_T)^2 + 2(1-x_O)(1-x_T)^3]/3 \ ,$$
$$p(1Al) = [2x_O(1-x_O)(1-x_T)^2 + 2x_T(1-x_O)^2(1-x_T) + 2x_O(1-x_T)^3 + 6x_T(1-x_O)(1-x_T)^2]/3 \ ,$$
$$p(2Al) = [4x_O(1-x_O)x_T(1-x_T)^2 + x_O^2(1-x_T)^2 + x_T^2(1-x_O)^2 + 6x_Ox_T(1-x_T)^2 + 6x_T^2(1-x_O)(1-x_T)]/3 \ ,$$
$$p(3Al) = [2x_O^2x_T(1-x_T) + 2x_T^2x_O(1-x_O) + 6x_Ox_T^2(1-x_T) + 2x_T^3(1-x_O)]/3 \ ,$$
$$p(4Al) = [x_O^2x_T^2 + 2x_Ox_T^3]/3 \ . \qquad \text{Equation S11}$$

Again, in the limit of $x_O = x_T$, Equation S11 can be seen to be equivalent to Equation S1. Figure 9b of the main text shows the integrated intensities expected using Equation S1, whereas Figure 9c shows the intensities expected from Equation S11.

S8. References

S1. M. Amri, S. E. Ashbrook, D. M. Dawson, J. M. Griffin, R. I. Walton, S. Wimperis *J. Phys. Chem. C*, 2012, **116**, 15048.

S2. K. J. Pike, R. P. Malde, S. E. Ashbrook, J. McManus, S. Wimperis *Solid State Nucl. Magn. Reson.*, 2000, **16**, 203.

S3. D. S. Cook, J. E. Hooper, D. M. Dawson, S. E. Ashbrook, J. M. Fisher, D. Thompsett, R. I. Walton *Inorg. Chem.*, 2020, **59**, 3805.

S4. D. M. Dawson, L. E. Macfarlane, M. Amri, R. I. Walton, S. E. Ashbrook *J. Phys. Chem. C*, 2021, **125**, 2537.

S5. S. E. Ashbrook, D. McKay *Chem. Commun.*, 2016, **52**, 7186.

S6. R. Grau-Crespo, S. Hamad, C. R. A. Catlow, N. H. de Leeuw *J. Phys.: Condens. Matter*, 2007, **19**, 256201.

S7. C. Schott-Darie, H. Kessler, M. Soulard, V. Gramlich, E. Benazzi *Stud. Surf. Sci. Catal.*, 1994, **84**, 101.
